# Diagnosis and Treatment of *Plasmodium vivax* Malaria

**DOI:** 10.4269/ajtmh.16-0171

**Published:** 2016-12-28

**Authors:** J. Kevin Baird, Neena Valecha, Stephan Duparc, Nicholas J. White, Ric N. Price

**Affiliations:** 1 Eijkman-Oxford Clinical Research Unit, Jakarta, Indonesia.; 2 Centre for Tropical Medicine and Global Health, Nuffield Department of Medicine, University of Oxford, Oxford, United Kingdom.; 3 National Institute for Malaria Research, New Delhi, India.; 4 Medicines for Malaria Venture, Geneva, Switzerland.; 5 Mahidol Oxford Research Unit, Faculty of Tropical Medicine, Mahidol University, Bangkok, Thailand.; 6 Division of Global and Tropical Health, Menzies School of Health Research–Charles Darwin University, Darwin, Australia.

## Abstract

The diagnosis and treatment of *Plasmodium vivax* malaria differs from that of *Plasmodium falciparum* malaria in fundamentally important ways. This article reviews the guiding principles, practices, and evidence underpinning the diagnosis and treatment of *P. vivax* malaria.

## Diagnostic Practices for *Plasmodium vivax* Malaria

### Infection.

The diagnosis of *Plasmodium vivax* infection can be broadly categorized into three purposes: identification of clinical cases (passive case detection [PCD]), surveillance (active case detection [ACD]), and clinical trials. Each scenario brings distinct requirements, tools, and pitfalls for diagnosis of the infection.

#### Passive case detection.

The accurate diagnosis of vivax malaria in an acutely ill patient seeking routine care requires microscopy examination of a Giemsa-stained blood smear (microscopy), or use of an immunochromatographic cassette containing monoclonal antibodies to a *P*. *vivax* antigen (rapid diagnostic test [RDT]). Clinical signs and symptoms alone, though frequently used, can neither differentiate malaria infection from other causes of febrile illness, nor distinguish between *Plasmodium falciparum* and *P*. *vivax* or malaria caused by another plasmodia. Competent microscopy is typically more sensitive, specific, and informative (with respect to parasite count and stages present) than RDT. However, the sustainability of microscopy services challenges most health-care systems where endemic malaria occurs.^1^,^2^

##### Microscopy.

Standards for malaria microscopy training, certification, and practice are available from World Health Organization (WHO).^3^,^4^ Examination of at least 200 fields of a thick blood film under oil immersion magnification (×1,000) should be undertaken before a negative diagnosis is made. The limit of detection for expert microscopists is considered to be about 10–20 parasites/μL.^5^,^6^ Routine competent microscopy in clinical settings is considered unreliable below about 50 parasites/μL.

The density of parasitemia in patients with acute vivax malaria depends upon many factors, including naive versus a state of semi-immunity, age, delay in seeking treatment, self-treatment behavior before presentation, and likely a variety of host and parasite factors.^7^

The parasite density in *P*. *vivax* malaria is typically an order of magnitude lower than *P*. *falciparum* in most clinical settings where both these species occur, thus increasing the risk of false negative microscopy diagnosis with acute vivax malaria. Repeated blood film examinations, or increasing the number of microscopy fields to 300 or more in patients suspected of having malaria should be carried out before confidently reporting the patient as negative for malaria parasites.

The primary diagnostic threat in the clinical setting is poor sensitivity. Training of clinical microscopists should be aimed at maximizing parasite detection, even if that is at the cost of a lower level of specificity. In some settings where vivax malaria predominates and transmission of *P*. *falciparum* is falling, there may be particularly low sensitivity for *P*. *falciparum*.

In addition to relatively intensive training and certification for the microscopist, competent microscopy requires a clean and well-functioning light microscope, clean glass slides, immersion oil with appropriate optical properties, and fresh filtered reagents for Giemsa staining. In many settings of endemic malaria, these essentials represent substantial challenges that cannot be reliably sustained. Where the quality of microscopy services cannot be assured, the use of RDT is recommended.^8^

##### Rapid diagnostic tests.

The principle advantage of RDTs is their ease of use and sustainability in resource-challenged settings. RDTs are available from many commercial sources at relatively low cost (usually < US$1/test). Most of these kits are stable at ambient temperature storage for many months. WHO offers standards for training and certification in the use of RDT.^9^ The sensitivity and specificity of RDT varies greatly among commercial providers, and by species being diagnosed. In general, the kits perform better with *P. falciparum* infection than that with *P. vivax* (e.g., 74% versus 37% of test brands scored > 75% “parasite detection score” at a density of 200 parasites/μL, respectively^10^). However, among the several dozen tests evaluated, a dozen scored ≥ 90% on the parasite detection score for *P*. *vivax* and *P*. *falciparum* at 200 parasites/μL. These kits may be viewed as suitable for the diagnosis of acute vivax malaria by RDT (see Table 1 ). Some tests detect *P*. *falciparum* histidine-rich protein 2 in addition to a pan-genus antigen (lactate dehydrogenase [pLDH]). Reaction to both indicates presence of *P*. *falciparum*, either alone or mixed with any other species, whereas reaction to pLDH alone indicates absence of *P*. *falciparum* and presence of any other species, but does not distinguish among these. Other RDTs do offer a *P*. *vivax*-specific diagnosis employing aldolase antigen capture with satisfactory performance.^11^

#### Active case detection.

ACD is usually conducted through mass blood surveys with the intent of detecting asymptomatic carriers of infection for the purposes of either malaria control or measurement of prevalence of parasitemia in at-risk populations. Asymptomatic carriers typically have much lower parasitemias compared with those seeking medical attention for illness, and RDTs detect only a minority of these infected individuals. The majority of mass blood surveys (historically and today) are performed using microscopy. The growing realization of the importance of detecting low-level parasitemias has spurred development of alternative technologies such as loop-mediated isothermal amplification (LAMP) and polymerase chain reaction (PCR).^12^–^15^

##### Microscopy.

The process of microscopy for the purpose of ACD is similar to that described for PCD. A microscopist has some flexibility in the clinical setting regarding the degree of effort committed to an examination (e.g., > 200 fields and multiple smears examined for a single patient). However, in ACD, only one blood film is usually collected, and thus care must be taken to ensure that the same degree of diligence is applied for all samples collected.

##### Polymerase chain reaction.

The most widely applied and validated molecular diagnostic is a nested PCR amplification of small subunit ribosomal RNA gene sequences.^16^ The term nested refers to an initial amplification using primers of genus-wide sequences followed by primers of species-specific character to provide the definitive diagnosis. This technique, and other PCR-based approaches to diagnosis, requires relatively advanced laboratory equipment and technologically skilled execution. Typically, blood blots dried onto filter paper in the field are transported to a laboratory where extraction of DNA and its analysis by PCR is performed. It is also relatively expensive. The advantage of PCR is increased sensitivity to detect parasitemias up to three orders of magnitude lower (depending on blood volume sampled) compared with microscopy or RDT. Its absolute sensitivity in operational use is approximately 1.0 parasite/μL, increasing to 0.02 parasites/μL if > 0.25 mL of venous blood can be collected.

##### Loop-mediated isothermal amplification.

LAMP of parasite-specific DNA is a more recent technology more suited to ACD in endemic settings. The technique does not require expensive thermocyclers or gel electrophoresis, the readout being a visual color change in a small test tube. Recent evaluation of a commercially available kit (with *P*. *falciparum* and pan-genus-specific reagents) showed the technique to be comparable to standard nested PCR technique and superior to expert microscopy.^17^,^18^ The procedure can be completed in about 1 hour at the site of collection. A modified LAMP procedure, as well as standard nested PCR, was performed in the diagnosis of *P*. *vivax* in large field surveys in China.^19^

##### Serology.

Diagnostic techniques employing serological markers have been applied mostly to studies of protective immunity and epidemiology. Most published studies have applied techniques using antigens derived from synthetic peptide vaccine candidates rather than those that may be more informative of active or latent infection. Studies of the vaccine antigen–derived serological assays generally show very poor specificity with regard to diagnosis of active infection. Further work aimed at antibodies specific to acute infection, rather than prolonged protection against such infection, is required and should have the potential to identify populations at greatest risk of parasitemia.

### Glucose-6-phosphate dehydrogenase deficiency.

The diagnosis of glucose-6-phosphate dehydrogenase deficiency (G6PDd) is an important diagnostic procedure before initiating the radical cure of *P. vivax*, which currently requires administration of primaquine (PQ). PQ can cause serious hemolysis in patients with G6PDd, an inherited X-linked highly diverse disorder affecting about 400 million people. G6PDd occurs at a prevalence varying from < 1% to 30% among residents of endemic zones.^20^ There are many distinct variants of G6PD enzyme each with different degrees of compromise of its key metabolic function—the provision of reducing equivalents via nicotinamide adenine dinucleotide phosphate (NADPH) and reduced glutathione in sustaining redox equilibrium of the cytosol and protecting cell constituents from oxidative damage.^21^ In most genotypes, the mutant enzyme degrades more rapidly, rendering the older red cells the most deficient. The residual enzyme activity defines a clinically relevant and measurable phenotype for many variants.

Because the G6PDd is X-linked, it is either wholly absent or present (hemizygous) in males. In females, in contrast, it may be absent, homozygous, or heterozygous. Homozygosity in females is relatively rare (the square of the allele frequency), but heterozygosity is common. This is an important clinical and diagnostic problem due to the phenomenon of lyonization of X-linked genetic traits in women. This process results in two distinct populations of red blood cells, that is, those expressing defective or normal G6PD. The relative proportion of cells expressing abnormal enzyme averages 50% but ranges between 0% and 100%. In other words, heterozygous females may be G6PD normal or fully G6PD deficient; however, most have red blood cell populations presenting a mosaic of the two phenotypes. The clinical significance of this in the context of PQ toxicity is unclear, but challenges the widespread deployment of PQ to vulnerable populations.

#### Clinical diagnosis.

Ascertainment of G6PDd status in a clinical setting can involve the quantitative or qualitative measurement of G6PD activity in hemolysate, cytochemical microscopic examination of whole cells, or inference from genotyping extracted DNA using PCR technology.

##### Quantitative diagnosis.

Standardized commercially available kits may be used for the spectrophotometric determination of G6PD activity in enzyme units (U) per gram of hemoglobin (gHb). Normal values range approximately from 7 to 10 U/gHb (at 30°C).^22^ The genetic heterogeneity of phenotypically “normal” G6PD enzyme and varying environmental factors may account for the wide range of activity values.^23^ Patients presenting values less than 7 U/gHb are classified as G6PD deficient. G6PD activity is usually defined as a percentage of normal activity, as this provides an intuitive measure of likely vulnerability to hemolytic anemia. The “normal” denominator of the estimation is usually defined by the mean G6PD activity of most patients evaluated in any given patient population. Table 2 lists validated quantitative assays and their commercial providers.

In the quantitative assay, as in the qualitative assay, diagnosis of G6PDd in patients having recently suffered acute hemolytic anemia is problematic, since some patients may exhibit a normal phenotype as a consequence of the most vulnerable red blood cells having been removed and their replacement by young erythrocytes that have inherently higher G6PD activity levels. The effects of acute malaria on G6PD tests have not been evaluated.

Most G6PD tests require a separate Hb measurement, and this imposes additional complexity and costs. The reason of doing so, however, is the impact of Hb level on the qualitative measurement, for example, below about 8 g/dL Hb, G6PD activity measurements trend sharply higher, probably falsely so.^24^ G6PD activity measurements from anemic patients should not be considered reliable.

##### Qualitative.

Standardized commercially available kits allow a visual determination of G6PD phenotype. These all involve the enzymatic conversion of NADP+ to NADPH by G6PD, which may be visualized directly (using fluorescent lighting) or indirectly (using one of several azole dyes that change color in the presence of NADPH). The most commonly used and widely validated qualitative assays are listed in Table 2. An alternative option is the methemoglobin reduction assay.^25^,^26^

##### Cytology.

Cytological techniques can be used to measure the degree of X-inactivation by lyonization in heterozygous females. These methods differentially stain individual red blood cells and permit estimation of the proportion of cells expressing defective G6PD enzyme. A variety of techniques have been described,^27^ including a recent flow cytometry method of particular promise for clinical and research settings.^28^

##### Genetic.

The principal advantage of G6PD genotyping is a diagnosis un-confounded by the physiological variables affecting G6PD activity such as patients suffering acute hemolytic anemia or lyonization in heterozygous females. The primary disadvantage, however, is the uncertainty of a “normal” diagnosis when applying primers to selected known genotypes using standard PCR/restriction fragment length polymorphism (RFLP) techniques. The patient may be deficient but with a genotype not represented in the genetic analysis—a significant hazard with G6PD, which is a complex gene with over 200 known variants documented. New variants of G6PD are routinely found wherever sufficiently detailed investigations can be carried out.^29^,^30^ Although whole gene sequencing is certainly possible, it is currently impractical in a routine clinical sense due to its very large size (18.5 kb), complex structure (13 exons), and the occurrence of mutations all along its length. The most commonly applied methodology is PCR/RFLP and requires specific selection of a number of mutation-specific primers limited by practicality.

#### Point-of-care and survey diagnosis.

Most patients with vivax malaria receive care at home or in the community outside of a clinic. Even when patients attend a clinic or hospital with a laboratory facility, there is often no capacity for G6PDd diagnosis. Similarly, diagnosis of G6PDd for survey purposes also occurs in rural settings and often cannot accommodate relatively sophisticated laboratory techniques.

##### Point-of-care diagnosis.

There is currently no validated point-of-care (POC) diagnostic device for G6PDd that is practical to apply where most malaria patients live. The obstacles to such a test include cost, complexity, heat sensitivity, and the need for a cold chain to transport and store the kit. A commercial kit in development, however, shows promise in overcoming those issues: the CareStart G6PD™ (AccessBio, Somerset, NJ), which is affordable, easy to use, and is not sensitive to ambient temperature fluctuations. However, although it is capable of reliably detecting G6PD deficiency below 30% of normal activity,^31^,^32^ it is currently insensitive to milder deficiencies and most female heterozygotes, a limitation of other qualitative tests. Although promising, CareStart G6PD™ requires more thorough assessment before it may be recommended for routine diagnosis of patients before PQ therapy.^33^

##### G6PDd survey.

Surveys to assess the prevalence of G6PDd are often undertaken where laboratory capacity is limited, the test readout being subjective and visually categorized into G6PD deficient, intermediate, or normal. A newer quantitative technique—WST8/1-methoxy-PMS—has been successfully used to survey populations with a quantitative enzyme activity readout. Dried blood blots collected in the field and kept refrigerated are returned to the laboratory for G6PD activity determination in a 96-well spectrophotometric assay format.^34^,^35^ The results correlate well with other standard techniques.

### Clinical trials.

Evaluation of the safety and efficacy of therapies for acute vivax malaria, which necessarily includes both blood schizontocidal and hypnozoitocidal therapies, employs diagnostics for both the infection and G6PDd. Clinical trials of experimental therapies often demand higher standards of diagnosis than may be applied in routine clinical care, to provide greater assurance of subject safety and the precision of efficacy estimates.

#### Diagnosis of *P. vivax* and G6PDd for blood schizontocidal efficacy.

Trials of blood schizontocidal therapies typically only enroll patients with parasitemias above a certain threshold, to increase the likelihood that the associated fever is due to the infection. When parasitemias exceed 500/μL, almost any diagnostic technique will suffice. However, microscopy remains the method of choice for staging the parasite and documenting the parasite clearance.

Screening for G6PDd is required for PQ therapy in trials and experimental blood schizontocide, which do not have a demonstrated safety profile in G6PD-deficient patients.

#### Diagnosis of *P. vivax* and G6PDd for hypnozoitocidal efficacy.

The methodology of anti-relapse efficacy trials is challenging, but broadening enrollment criteria may be warranted to include those with subpatent infections. In this context, the use of highly sensitive diagnostic techniques such as LAMP may help to discriminate patient risk groups at enrollment. It is important to identify patients with G6PD variants known to be at high risk of severe hemolysis. Historically, the NADPH spot test (which identifies patients with < 30–40% of normal activity) has been used for this purpose, including a series of trials of PQ as primary prophylaxis in the past decade where subjects received large cumulative doses of this drug over prolonged periods.^36^ Although valid concerns may be raised regarding insensitivity to milder variants and female heterozygotes, there are no documented cases of acute severe hemolytic anemia following PQ therapy and a classification as normal by the NADPH spot test. At least in the specific instance of PQ, this record of safe use with NADPH spot test screening argues in favor of good safety of this technique or those of similar diagnostic performance. Nonetheless, direct evidence of safety in this practice is lacking.

#### Diagnosis of recurrent parasitemia.

Clinical efficacy is defined by the clearance and recurrence of parasitemia. In most patients these occur at relatively low levels of patency, and thus expert microscopy is crucial in the assessment of blood films. In contrast to routine clinical microscopy, the microscopist serving clinical trials must undergo rigorous certification to minimize false-positive outcomes, which have potential to significantly underestimate clinical efficacy.^37^ False negative (intolerable in clinical settings) is less important in the detection of recurrent parasitemia in asymptomatic individuals being routinely followed up. Confirmation of the microscopic diagnosis by PCR techniques is often performed weeks or months later. Though microscopy lacks sensitivity relative to PCR techniques, it is far more unambiguous, whereas false-positive PCR by contamination is a well-known pitfall.

## Treatment Practices for *P. vivax* Malaria

The goals of antimalarial treatment in *P*. *vivax* are to reduce the immediate risk to the host, eradicate peripheral asexual parasitemia, prevent the recurrent infection, and interrupt the cycle of transmission.^38^ The ability of *P*. *vivax* to form dormant liver stages (hypnozoites) capable of causing relapsing infections weeks to months after the initial blood-stage infection, provides a major challenge to the complete eradication of parasites from the body. Since no single drug achieves all of these aims, a combination of antimalarials is required targeting a variety of specific key elements of the parasite life cycle.^1^

### Treatment of asexual erythrocytic stages of *P. vivax*.

#### Treatment of uncomplicated vivax malaria.

In areas where *P. vivax* is known to be chloroquine (CQ) sensitive, the WHO recommends 3 days of CQ or an artemisinin combination treatment plus 2 weeks of PQ (provided the affected individual is not G6PD deficient).^39^ CQ remains a first-line treatment in most parts of the world due to its wide availability, low cost, and long terminal elimination half-life. However, in co-endemic malarious areas, this necessitates a separate treatment approach for *P*. *falciparum* and *P*. *vivax*.

Most commonly used antimalarial drugs are also active against the asexual stages of *P*. *vivax*, the exception being the antifolates, which act slowly,^40^ and are vulnerable to the rapid development of drug resistance.^41^,^42^ Mefloquine,^43^ atovaquone + proguanil,^44^ halofantrine,^45^ piperaquine,^46^ artesunate,^47^,^48^ and pyronaridine,^49^ all show good efficacy against chloroquine-resistant (CQR) *P*. *vivax* in clinical trials.

Artemisinin combination therapies (ACTs) are the treatment of choice for CQR *P*. *vivax*.^46^ WHO-recommended ACTs include artemether–lumefantrine, artesunate–amodiaquine, artesunate–mefloquine, and dihydroartemisinin (DHA)–piperaquine. A fifth ACT, pyronaridine–artesunate, has recently obtained a positive opinion from the European Medicines Agency for the treatment of *P*. *vivax* malaria, but it is not yet recommended by the WHO. Artemisinin in combination with effective partner drug have shown excellent cure rates in *P*. *vivax* infection.^50^ ACTs with partner drugs with longer elimination periods provide incidental suppressive prophylaxis against relapse for about a month, but relapse risk thereafter remains relatively high.

The deployment of an ACT-based strategy permits a unified policy for treating both *P*. *falciparum* and *P*. *vivax* infections, offering a pragmatic approach with operational efficiencies.^48^ A unified policy also decreases frequent issues of species misdiagnosis in routine practice. The rise and spread of CQR *P*. *vivax* has led to a number of countries adopting ACTs as first-line treatment for *P*. *vivax*. These include DHA–piperaquine in Indonesia and Cambodia, and artemether-lumefantrine in Papua New Guinea, Solomon Islands, Sudan, Namibia, South Africa, and Vanuatu.^51^ Unified treatment policy also infers anti-relapse therapy for *P*. *falciparum*, where *P*. *vivax* is sympatric (see Drug Development for *P. vivax* section).

#### Treatment of severe vivax malaria.

Severe and fatal vivax malaria has been reported from Indonesia,^52^,^53^ Papua New Guinea,^54^ India,^55^ and Brazil.^56^,^57^ The main manifestations are anemia and respiratory distress,^53^,^56^–^62^ although series of patients with coma, shock, and renal and hepatic dysfunction associated with vivax malaria have also been described.^55^–^57^,^61^,^62^ *Plasmodium vivax* is very sensitive to artemisinin and its derivatives. In the absence of comparative drug trials, physicians have tended to adopt a similar treatment approach for severe vivax malaria as for severe falciparum malaria,^1^ namely administration of parenteral artesunate, if unavailable, artemether, and if that is also not available then quinine, along with broad-spectrum antibiotic cover and supportive care.

Large-scale multicentered trials in patients in Asia and Africa have demonstrated clear superiority of intravenous artesunate over quinine in reducing case fatality rate in severe falciparum malaria.^63^,^64^ Intravenous artesunate also leads to a rapid clinical response in patients with severe vivax malaria,^53^,^61^ but there have been no randomized clinical trials in severe *P. vivax* malaria.

Specific antimalarial treatment recommended in severe vivax malaria includes the following in order of preference:

• Artesunate: 2.4 mg/kg body weight, intravenously or intramuscularly given on admission (time = 0), then at 12 and 24 hours, and then once a day. This is the treatment of choice.
• Artemether: 3.2 mg/kg body weight, intramuscularly given on admission, then 1.6 mg/kg body weight per day.
• Quinine: 20 mg quinine salt/kg body weight on admission (intravenous infusion in 5% dextrose/dextrose saline over a period of 4 hours) followed by maintenance dose of 10 mg/kg body weight 8 hourly (maximum infusion rate 5 mg salt/kg/hour).

Parenteral antimalarials should be administered for at least 24 hours. Once the patient can accept oral therapy, full course of oral ACT should be given to the patients. Full details are available in the latest Malaria Treatment Guidelines.^1^

#### CQR *P. vivax*.

**Figure 1.**
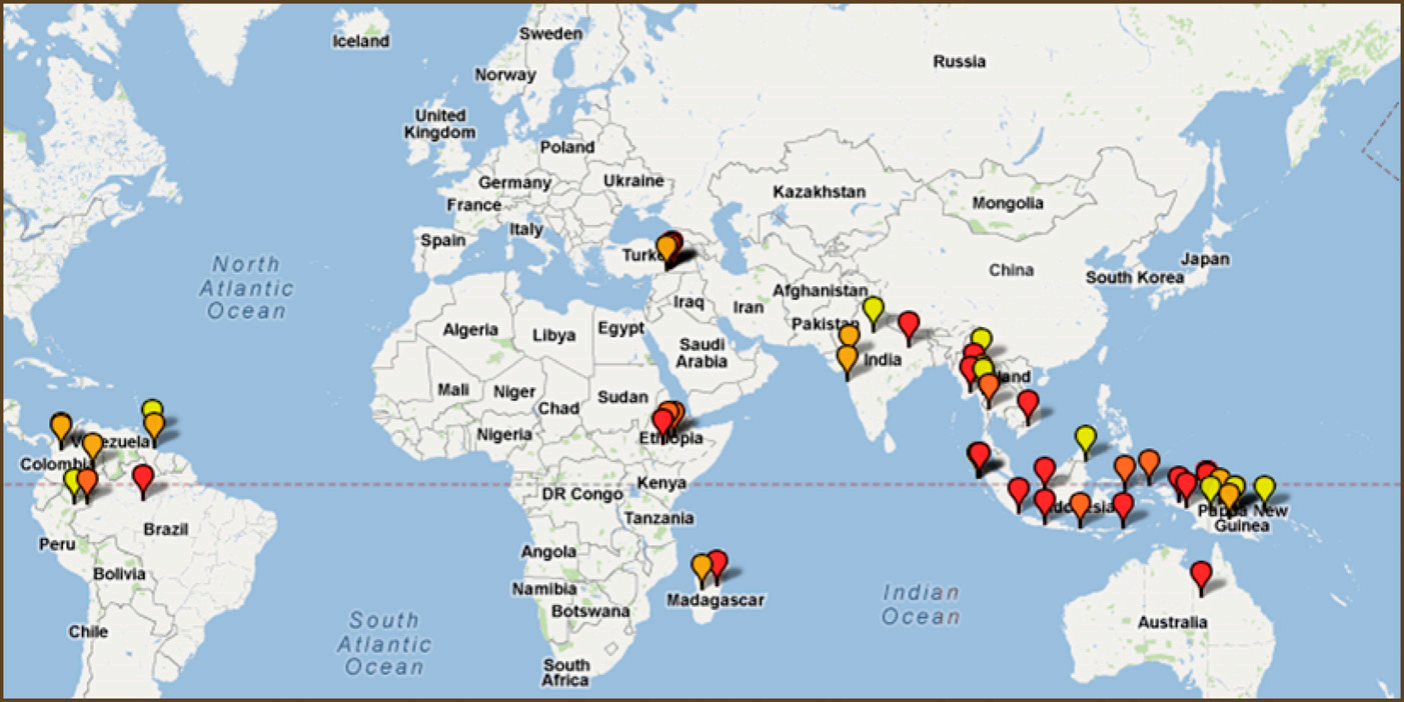
Clinical reports of chloroquine-resistant *Plasmodium vivax*. Red icons highlight areas of chloroquine-resistant parasites defined by greater than 10% recurrence (and lower 95% confidence interval [CI] > 5%) by day 28 with or without measurement of chloroquine drug concentration; dark orange diamonds locate area suggestive of resistance as defined by 5–10% recurrence by day 28, confirmed with adequate chloroquine drug concentrations; light orange icons locate sites of possible resistance as defined by less than 5–10% recurrence by day 28 but with lower 95% CI < 5% by day 28, without drug concentrations. Yellow icons represent case reports. Details of clinical trials are provided in Supplemental Annex A.

The first reports of CQR *P. vivax* were published in 1989 from Australian travelers to Papua New Guinea,^65^ and in 1991 as an endemic problem in Indonesia,^66^ 30 years after the documentation of CQR *P. falciparum*. Although intrinsic differences in the transmission dynamics between these two species may account for much of the time lag, it is likely that this also reflects the inherent complexity of defining antimalarial treatment efficacy of *P. vivax*.^67^ High-grade CQR *P. vivax* has been documented on the island of New Guinea, where patients treated with CQ have been observed to have early clinical deterioration requiring hospitalization, delayed parasite clearance, and early recurrent parasitaemia.^46^,^68^,^69^ Evidence for declining CQ efficacy against *P. vivax*, albeit to a lesser degree, has been reported from across the vivax-endemic world (Figures 1

**Figure 2.**
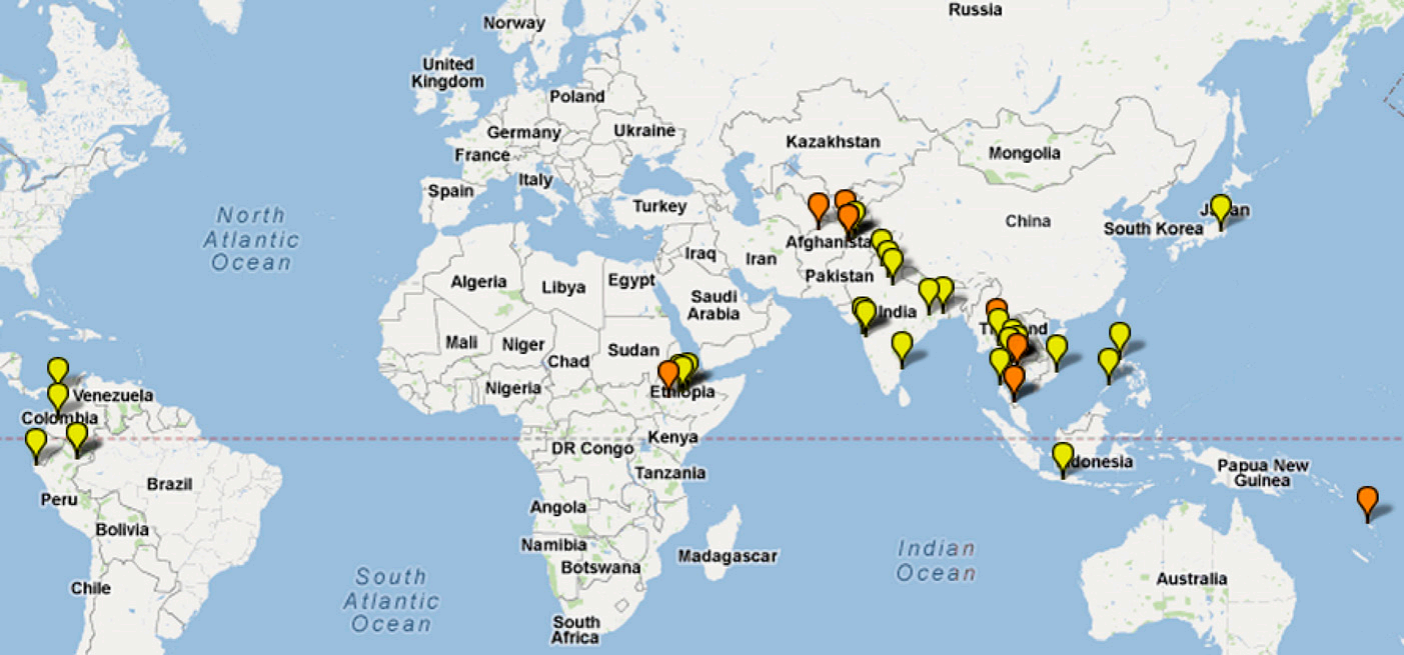
Clinical reports of chloroquine-sensitive (CQS) *Plasmodium vivax*. CQS was defined as < 5% recurrence by day 28, no early administration of primaquine, and all patients from symptomatic clinical presentation. Yellow icons represent studies before 2007, orange icons studies between 2007 and 2012. Details of clinical trials are provided in Supplemental Annex A.

–3

**Figure 3.**
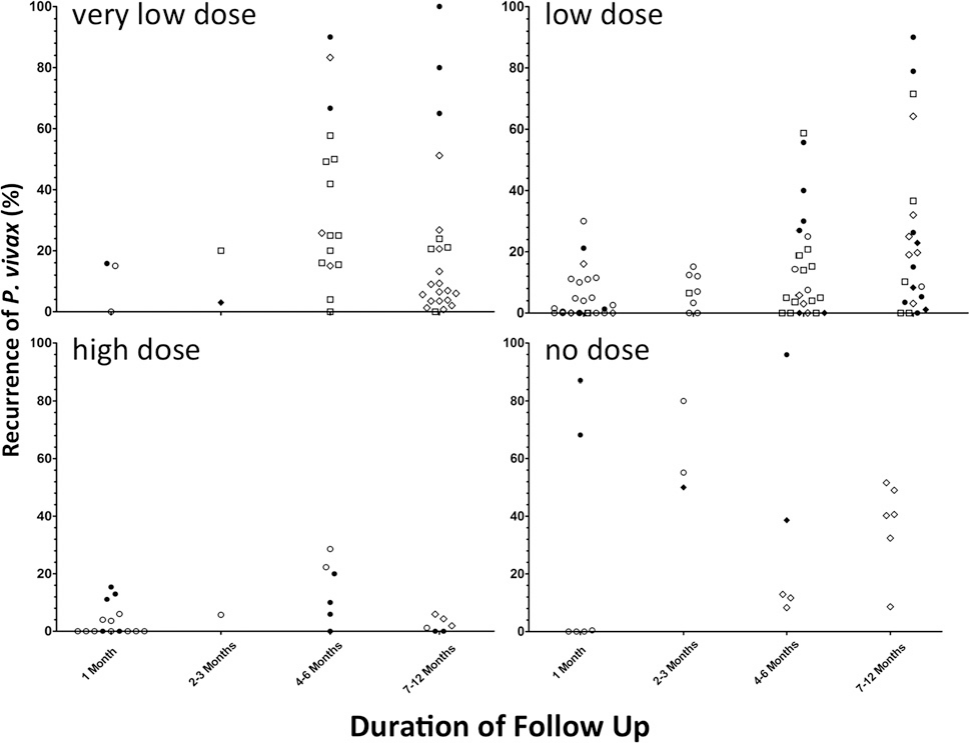
Risk of recurrence at the end of the study following very low-dose primaquine (PQ) (total dose ≤ 2.5 mg/kg), low-dose PQ (total dose > 2.5 mg/kg to < 5.0 mg/kg), high-dose PQ (total dose > 5.0 mg/kg). Indonesia and Papua New Guinea [closed circles]; Thailand and Vietnam (open circles); South and Central America (open squares); Indian subcontinent, Middle East, and Horn of Africa (open diamonds); and Korea and China (closed diamonds). The U.S. studies of induced malaria and returning soldiers are categorized according to origin of infecting strain.

).^70^–^72^

##### In vivo efficacy.

The WHO's protocols for the evaluation of antimalarial efficacy focus primarily on the treatment of *P. falciparum*. These guidelines have undergone extensive revision over the last 20 years, the more recent versions extending their scope to investigate the therapeutic efficacy against *P. vivax* infections. Current guidelines for assessing CQ recommend supervised treatment and follow-up for a minimum of 28 days, accompanied by measuring whole blood CQ and desethylchloroquine level at the day of failure. Recurrent infections during this period presenting with whole blood CQ plus desethylchloroquine concentration exceeding 100 ng/mL are considered as resistant irrespective of whether they are relapse, recrudescence, or reinfection.

A major confounding factor in interpreting clinical drug efficacy against *P. vivax* is an inability to distinguish reliably between relapse, recrudescence, or reinfection. A variety of methodologies have been developed for *P. vivax* with as few as three polymorphic markers proving to be sufficient to discriminate homologous from heterologous infections.^73^–^76^ However, recurrence of *P. vivax* genetically identical to the pretreatment isolate can occur from either a true recrudescence of the initial infection or a relapse from hypnozoites generated from the prior blood-stage infection^74^,^77^; unsurprisingly molecular methods are unable to distinguish between these alternatives. Relapses are also commonly genetically heterologous. The confounding effect of relapsing infections varies considerably between geographical locations, both for the absolute risk of relapse and the timing at which these occur. In equatorial regions, 50–80% of patients can have a relapse starting within 3 weeks of the initial infection (if a rapidly eliminated drug is used for treatment), whereas in patients infected by temperate strains, the risk of relapse may fall to 5–20%, recurrences occurring many months after the initial infection.^78^ Major impediments to defining CQ resistance and common causes of misdiagnosis of CQ resistance and susceptibility come from multiple aspects (Table 3 ).

**Figure 4.**
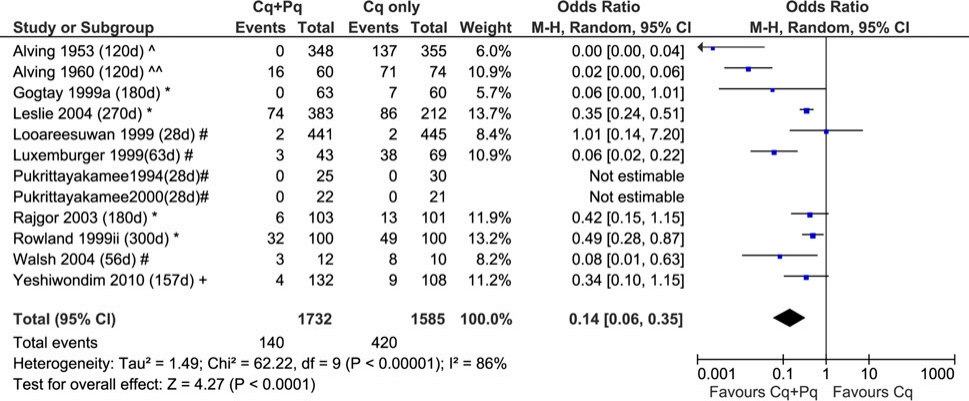
Forest plot of the effectiveness of low-dose primaquine in studies with a control arm. * Indian subcontinent, ^ United States (Korea), ^^ United States (Chesson), # Thailand, ## Indonesia, + Ethiopia.

A recent review of the literature identified 135 prospective clinical trials of antimalarial efficacy against the erythrocytic stages of *P. vivax* monoinfection published between 1980 and 2013, of which full manuscripts were available for 124 studies. There have also been 28 case reports of CQ resistance. A complete list of these extracted data is available in Supplemental Annex A. In total there have been 121 treatment arms documenting the efficacy of CQ (enrolling 13,878 patients) and 21 treatment arms assessing the clinical efficacy of ACTs (artemether–lumefantrine, eight; DHA–piperaquine, seven; sulfadoxine/pyrimethamine, three; and one each for amodiaquine–artesunate, pyronaridine–artesunate, and artemisinin–naphthoquine). CQ efficacy has been quantified at 97 geographical locations, of which 47 (48%) revealed reduced potential susceptibility (Figure 1 and Supplemental Annex A). Numerous studies demonstrate the great therapeutic benefit of primaquine therapy at low (Figure 4

) or high (Figure 5

**Figure 5.**
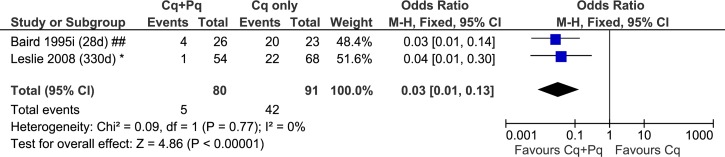
Forest plot of the effectiveness of high-dose primaquine in studies with a control arm. * Indian subcontinent, ## Indonesia.

) dosing when combined with chloroquine versus chloroquine alone, i.e., odds of recurrence of 0.14 (0.06–0.35) or 0.03 (0.01–0.13), respectively.

##### Ex vivo and molecular assays.

The in vitro assessment of parasite drug susceptibility has proven to be very useful in the investigation and mapping of drug-resistant *P. falciparum*; however, the development of similar tests in *P. vivax* is more challenging. Unlike *P. falciparum*, the parasite preferentially invades young red blood cells, limiting its reproductive capacity and ability to adapt in continuous in vitro culture.^79^,^80^ Without culture adaptation, the ex vivo assessment of drug susceptibility in *P. vivax* field isolates has been limited to clinical samples derived directly from the human host and subjected to short-term culture and drug exposure as exemplified by the schizont maturation test.^79^–^82^ The inability to sustain in vitro growth restricts analysis of field isolates to a single time point making an assessment of reproducibility difficult. Despite its limitations, the current schizont maturation assay has demonstrated utility in discriminating parasite populations with different degrees of CQR,^83^–^85^ characterizing drug susceptibility profiles of *P. vivax* to commonly used antimalarial drugs,^86^,^87^ and screening susceptibility to novel therapeutic agents.^88^–^93^ Development of methods capable of sustaining *P. vivax* in continuous in vitro culture will transform the current ex vivo assay, accommodating cryopreservation of field isolates to reduce the reliance on the analysis of fresh isolates.

The identification of a molecular marker of CQ resistance in *P. vivax* remains elusive. Early studies failed to show a strong correlation between *pvcrt-o* and the CQR phenotype,^84^,^94^,^95^ although more recently interest has focused on the transcription level of *pvcrt-o* and its overexpression.^96^ A sequence polymorphism in *pvmdr1* conferring Y976F has been reported in a number of studies and may correlate with CQ resistance^84^,^97^; however, since CQ resistance can occur in isolates with wild type *pvmdr1*, *pvmdr1* mutations are likely to be at best minor determinants of CQ susceptibility.^84^,^98^,^99^

### Treatment of liver stages of *P. vivax*.

#### Determinants of efficacy.

For over 60 years, clinicians, policy makers, and patients have relied on PQ, an 8-aminoquinoline, for the radical cure of *P. vivax*. Primaquine is the only licensed antimalarial with proven hypnozoitocidal activity, but can result in significant hemolysis particularly in those with G6PDd.^100^,^101^ In view of the risk of adverse reactions, PQ dosing strategies are influenced more by concerns over toxicity than by their absolute efficacy. These concerns are particularly important in poorly resourced settings where routine G6PDd testing is often unavailable.

The predominant determinant of therapeutic efficacy appears to be the total dose of PQ administered rather than the daily dosage or duration of therapy.^102^ In an attempt to reduce potential toxicity, the WHO guidelines for the radical cure of vivax malaria currently recommends the use of a daily dose of 0.25 mg/kg/day (3.5 mg/kg total dose) PQ taken with food once daily for 14 days, coadministered with CQ or ACT depending on CQ sensitivity in the region.^103^ In southeast Asia and Oceania, the same guidelines recommend a higher daily dose of 0.5 mg/kg (7.0 mg/kg total dose) in view of the high risk of relapses.

A recent review of the published literature identified 87 clinical trials presenting data on 59,735 patients enrolled in 156 treatment arms^104^; the extracted data from these studies are presented in Supplemental Annex B. The median rate of recurrence in the 44 studies of very low dose of PQ (total dose ≤ 2.5 mg base/kg) was 25% (range: 0–90%) at 4–6 months, compared with 6.7% (range: 0–59%) in the 82 studies of low-dose PQ (total dose > 2.5 mg/kg to < 5.0 mg/kg). High-dose PQ regimens (total dose > 5.0 mg/kg) were assessed in 28 treatment arms, and were associated with a median recurrence rate of 0% (range: 0–15%) at 1 month. However, comparisons between dosing regimens need to be interpreted with caution, since they are confounded by the marked heterogeneity in study design, dose regimens, and idiosyncrasies of the endemic setting notably variable rates of reinfection.

The frequency and the timing of relapses are determined by sporozoite inoculum and parasite relapse phenotype, and this results in huge regional variation in the absolute risk of relapse independent of PQ efficacy.^78^ A better indication of PQ efficacy requires comparison with a control arm in which patients receive no PQ, thus controlling for background reinfection and relapse patterns.^105^,^106^ There have been 18 published studies with control arms. Of these, the effectiveness of a very low-dose PQ regimen was no different from patients who did not receive PQ, whereas for the low-dose regimens, a significant difference was reported in half of the studies (overall odds ratio [OR]: 0.14, 95% confidence interval [CI]: 0.06–0.35, *P* < 0.001). Two studies enrolling 171 patients demonstrated high effectiveness of high-dose PQ compared with a control arm (OR: 0.03, 95% CI: 0.01–0.13, *P* < 0.0001). Two recent studies from Indonesia documented good efficacy (> 95%) against relapse in *P. vivax* among soldiers followed for a year (without risk of reinfection) after directly observed high-dose PQ therapy either following^107^ or concurrent with ACT.^108^

Current guidelines recommend a 14-day course, but such prolonged courses of treatment can result in significant problems with adherence.^109^,^110^ Poor adherence to a 14-day course of unsupervised PQ is likely to have a major impact on its public health benefit. One study showed comparable efficacy of the 14-day course following strong health messaging and clear instructions for completion of the course in Afghan refugees in Pakistan.^111^ Short-course, high-dose regimens have potential to increase patient adherence, and thus effectiveness,^112^ but appropriately powered prospective multicentered randomized controlled trials are needed to assess the tolerability, safety, efficacy, and effectiveness of such alternative radical curative regimens against current best practice.

#### Primaquine-resistant *P. vivax*.

There is no evidence of resistance to therapeutic doses of PQ by hypnozoites of *P. vivax*. This may reflect the absence of the phenomenon or the extraordinary difficulty of gathering unambiguous evidence of it. Ingram and others^113^ described the process of doing so in assessing a patient in New Zealand who relapsed repeatedly despite high-dose PQ therapy. Other possible causes of therapeutic failure must be ruled out, principally 1) insufficient PQ quality, dose, adherence, absorption, or metabolism; 2) recrudescence due to CQ resistance, or inadequate therapy per above; and 3) reinfection after therapy. A loss-of-function cytochrome-P450 2D6 genotype resulting in inadequate metabolism of PQ is an important cause of therapeutic failure, as occurred among two *P. vivax* experimental challenge subjects.^114^

#### Safety considerations.

The most common serious adverse effect (SAE) following PQ is intravascular hemolysis associated with the passage of dark or black urine and mild jaundice. There are two potentially lethal consequences; life-threatening anemia and acute hemoglobinuric renal failure. From 69 studies and additional case reports (excluding data from mass treatment campaigns) that evaluated adverse events to PQ, no SAEs were reported in G6PD-normal individuals, with the possible exception of one psychotic reaction in an individual with undetermined G6PD status.^115^ The 191 SAEs that were reported were in 25 individuals who were assumed to be G6PD deficient and in 166 with proven G6PDd (139 of these were described in case reports). The incidence of SAEs in the known G6PD-deficient group was 11.2% (27/241). Of all SAEs, 11.5% occurred after a probable overdose of PQ (mainly in children), 75.9% with radical curative regimens of 15 or 30 mg daily for vivax malaria, and 12.6% after administration of 30 or 45 mg PQ in weekly prophylactic or radical curative regimens, or as a single-dose gametocytocide.

In total 15 deaths associated with PQ have been reported over the past six decades, of which 13 were from severe haemolysis.^115^ No deaths were reported from the mass radical treatments (MDA) in Jiangsu, China (> 28 million treated) or from the combined experience in Azerbaijan, Afghanistan, Tajikistan, and Democratic People's Republic of Korea (> 8 million). G6PD screening was not performed in these MDAs but hemolysis was anticipated and was observed, and so education was provided and health services reinforced during the drug administration. The reported deaths occurred mainly in countries with a minority of the global malaria burden, which raises concern about the generalizability of this estimate. G6PDd is known to be very rare in northern China and the Koreas for example. The nations of greatest risk—with significant burdens of *P. vivax* and highly prevalent Mediterranean or Mediterranean-like severe G6PDd variants^20^—often have poor pharmacovigilance and mortality reporting systems. Further, patients not surviving PQ therapy may often be presumed to have died as a consequence of malaria, as occurred in at least two cases reported from Brazil.^116^,^117^ Primaquine therapy against relapse certainly has the potential to cause lethal hemolytic anemia, but the incidence of such events cannot be known in the absence of evidence gathered by deliberate and direct surveys.

**Figure 6.**
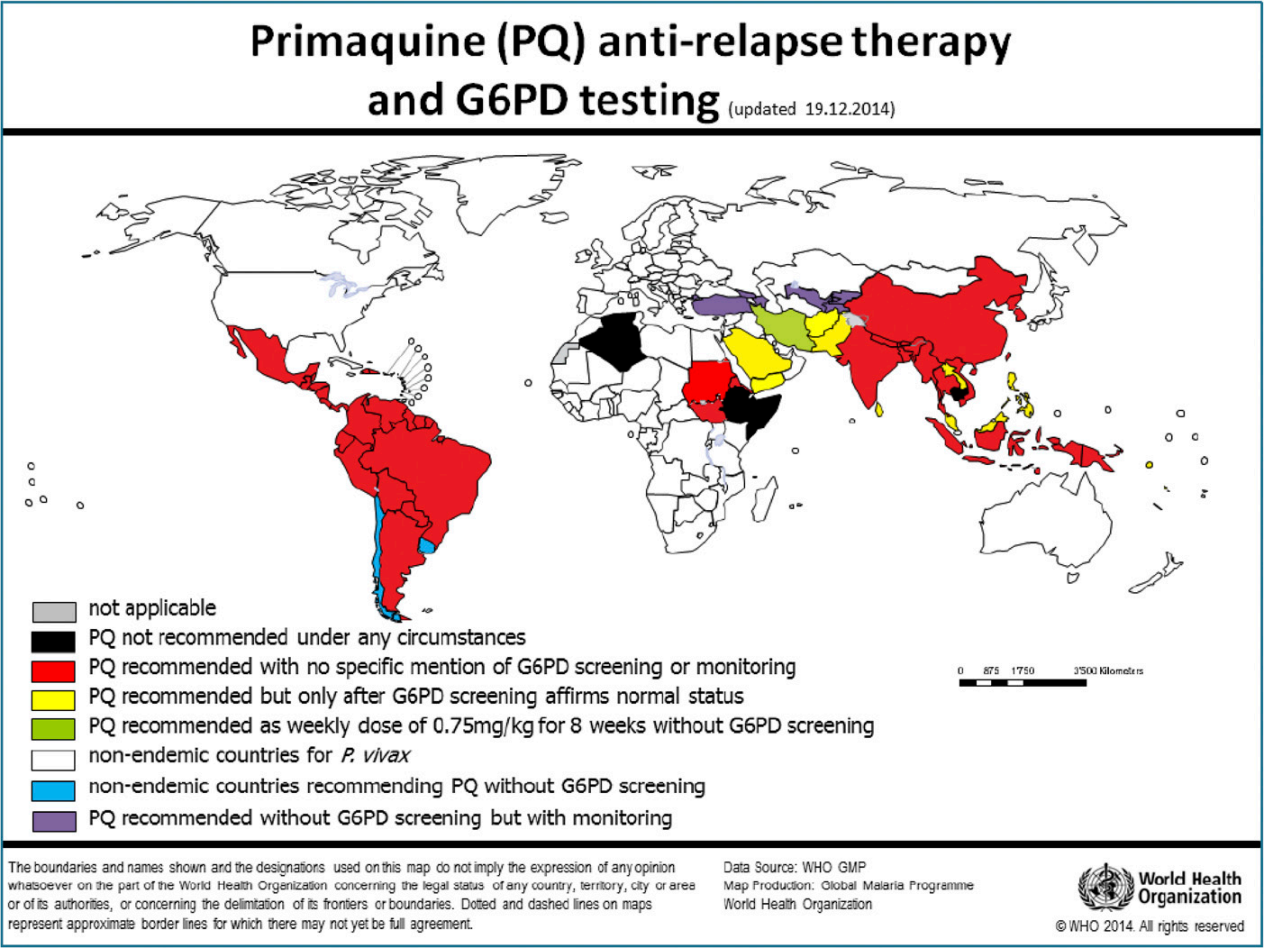
National malaria treatment guidelines and recommendations concerning primaquine anti-relapse therapy and G6PD screening, reprinted with permission of World Health Organization.

Primaquine is underused. Several studies highlight that even in areas where PQ is recommended, in practice it is prescribed rarely.^118^ Some endemic countries recommend the use of PQ with warnings on its potential toxicity in G6PD-deficient patients, but most do not (see Figure 6

).^33^ The lack of an available POC G6PD test means that the practitioner who prescribes a radical curative course of PQ risks inducing potentially serious hemolysis if the patient is G6PD deficient. POC testing to identify those at hemolytic risk would greatly enhance safe deployment and thus use of PQ. It is also important to balance this risk (borne entirely by those patients who are G6PD deficient) with the benefits derived by all treated patients; each *P. vivax* episode is associated with malaria-related hemolysis and dyserythropoiesis and a cumulative risk of anemia. This contributes to impaired development, school and work performance, and in high-transmission areas to anemia-related deaths. Without prolonged monitoring over repeated episodes of malaria, the benefits of radical cure may not be appreciated.

If there is no available G6PD POC test, it is difficult to generalize on the correct approach to patient management. The risk versus benefit weighing depends on the prevalence and severity of G6PDd in the area, the degree of anemia, and the availability of blood transfusion (i.e., the risks) as well as the probability of relapse and the likely dose regimen required (the benefit). In some circumstances the assessment may favor withholding PQ, and in others it may favor starting the radical curative regimen after educating the patient about the possible risks and informing the patient that they should stop the drug if they fail to recover within 3 days, become ill, or their urine becomes red or black.

#### Known G6PDd.

The evidence of PQ safety in known G6PD-deficient subjects or patients is limited to just four of the many dozens of known G6PD variants (A−, Mahidol, Mediterranean, and Viangchan) and less than a couple dozen subjects. In general, variants with > 5% residual G6PD activity showed relatively mild and self-limiting hemolysis, whereas those with < 5% activity showed severe hemolysis. There is insufficient evidence on which to guide PQ therapeutic decisions based on known or suspected degree of G6PD enzyme impairment. Any diagnosis of G6PDd in a male hemizygote should be considered indicative of risk of serious harm and prompt withholding standard daily PQ therapy against relapse.^33^ An alternative regimen, 0.75 mg/kg weekly for 8 weeks was demonstrated in the early 1960s to cause only slight hemolysis in otherwise healthy African–American men having A− G6PDd.^119^ This regimen became widely recommended for anti-relapse therapy in G6PD-deficient patients, as well as underpinning an earlier recommendation for the same dose administered only once as gametocytocidal therapy for *P. falciparum* malaria.^120^,^121^ However, the safety of the weekly regimen in non-A− variants was not established.^122^,^123^ Only very recently were 18 Cambodian men with G6PDd (17/18 with Viangchan variant) and acute *P. vivax* malaria given the 0.75 mg/kg PQ dose weekly for 8 weeks.^124^ All hemolyzed steeply after the first dose, but not dangerously, except for one subject who required hospitalization and transfusion. Subsequent weekly dosing showed only slight hemolysis. All known G6PD-deficient men receiving the weekly regimen of PQ should be clinically monitored after the first and second dose of PQ for assurance of safety.

Females present a more complex and difficult therapeutic problem. Homozygous females carry two mutant X alleles and phenotypically mirror hemizygous males in having 100% defective red blood cell populations. Female heterozygotes possess both wild type and mutant G6PD phenotypes, expressed as mosaicism in their red blood cell populations. Random inactivation of one or the other X chromosome during embryonic development results in variable proportions of wild type and mutant phenotypes among their red blood cells (a process called lyonization).^125^ The net G6PD activity measured in the whole blood of heterozygotes thus represents the average of two distinct subpopulations of red blood cells. The vulnerability of the mutant subpopulation to PQ-induced hemolysis may be presumed to be complete. In other words, a female testing at 50% of normal G6PD activity will have approximately 50% of her red blood cells exposed to possible destruction by PQ.^33^ The therapeutic problem arises with G6PD screening applying a 30% of normal activity threshold for identifying G6PD deficients by qualitative screening^33^: females testing as normal with > 30% G6PD activity may nonetheless be vulnerable to steep and threatening hemolysis with PQ therapy. This imposes the necessity of caution and clinical monitoring for all females screened as normal by standard qualitative G6PD screening tests for at least the first week of therapy.

#### Radical treatment in special groups.

##### Children.

There are relatively few data on the safety of PQ in young children, but there is no indication that toxicity in children differs from that in adults. Thus PQ is recommended in young children above 6 months of age, and not in infants (because of lack of data, rather than evidence of toxicity).^1^ However, since G6PDd is specifically associated with neonatal jaundice and kernicterus, PQ should definitely be avoided in the first month of life (and in any case is unnecessary as there are no hypnozoites in congenital vivax malaria). Tablets containing doses less than 7.5 mg (base) of PQ are often unavailable, so accurate dosing is difficult, which leads to both under- and overdosing. Since the mg/kg dosing variation is greatest in young children, studies are needed as a priority to assess PQ safety in these vulnerable age groups.

##### Pregnant women.

Primaquine is contraindicated in pregnancy because of the unknown G6PD status of the fetus and thus the risk of inducing intrauterine hemolysis. Excretion of PQ in breast milk is not known so caution should be exercised and preferably PQ should not be given. If PQ is prescribed to a lactating mother, the baby needs to be observed closely.

##### Human immunodeficiency virus–positive patients.

There is no specific information on the safety of PQ in this important patient group. Primaquine has been used as a second-line treatment (with clindamycin) of *Pneumocystis jirovecii* infections without apparent complications.

##### Drug interactions.

Primaquine is metabolized by monoamine oxidase to the biologically inert, but slowly eliminated, carboxyprimaquine, and via CYP_450_ (predominantly 2D6) to reactive intermediates that mediate both the antimalarial effects and hemolytic toxicity. Theoretically, inhibitors such as quinidine, ketoconazole, paroxetine, and fluoxetine may reduce toxicity, but these predictions require further study. Historic examples of likely drug–drug interactions have occurred between pamaquine (a PQ precursor) and mepacrine (structurally similar to CQ) impacting safety, and between quinine and CQ impacting efficacy against relapse.^126^

## Drug Development for *P. vivax*

Outside of Africa, *P. falciparum* and *P. vivax* commonly occur together; mixed infection is very common, and many patients with falciparum malaria harbor *P. vivax* hypnozoites that commonly cause relapse 3–8 weeks after the acute illness. The emergence of CQ resistance in many endemic regions, along with > 50% prevalence of hypnozoites of *P. vivax* in patients diagnosed and treated for *P. falciparum*, provides a strong rationale for a unified policy for therapy of uncomplicated malaria of any species.^127^,^128^ Hence, many of the schizontocidal drugs under development are now routinely tested against both dominant species of plasmodia.^129^ In general, those with high potency against multidrug-resistant *P. falciparum* are also efficacious against *P. vivax*.^129^ The greatest challenge for achieving radical cure is the development of new agents with hypnozoitocidal activity, which can be administered safely and effectively in short-duration treatment regimens.^130^

### Tafenoquine.

Tafenoquine (TQ; SB-252263 and WR238605), is an 8-aminoquinoline with in vivo anti-hypnozoite activity. The mechanism of action of TQ, as for all the 8-aminoquinoline class of drugs, is unknown. TQ has a long half-life enabling radical cure treatment as a single dose.^131^ TQ has been shown to be well tolerated and efficacious in vivax malaria radical cure in patients without G6PDd. A phase III study is underway with at least 600 subjects without G6PDd randomized to one of three treatment arms, TQ/CQ, PQ /CQ, or CQ alone, in a 2:1:1 ratio. This will assess the superiority of TQ/CQ over standard doses of CQ alone.

Phase I and IIb studies have been conducted and phase III efficacy and safety studies are ongoing.^132^ The phase I safety study investigated the hemolytic potential of TQ in healthy subjects with G6PDd, as well as the safety and tolerability of TQ in acute *P. vivax* malaria patients with G6PDd. The objective of the phase I dose escalation study was to determine the safety (hemolytic potential) and tolerability of TQ in G6PD-deficient healthy subjects without the influence of disease-related confounding factors. For the initial dose escalation phase, G6PD-deficient heterozygous female healthy volunteers with enzyme activity range between 40% and 60% of the site median normal value were recruited.

### Blocking 8-aminoquinoline hemolytic toxicity.

Current options for radical cure are dependent upon the 8-aminoquinolines. Compounds that are protective against the oxidative damage from PQ or TQ have potential for safe deployment and accessibility to radical cure. Novel assays exist to screen for such products, for instance an in vivo immunodeficient murine model transfused with human G6PD-deficient blood.

#### Methylene blue.

During the development of PQ in the late 1940s, one volunteer experienced deep hemolysis after a few 60 mg daily doses of the 8-aminoquinoline isopentaquine. Months later, however, when the same dose of isopentaquine was administered with an oral 500 mg dose of methylene blue (MB; methylthioninium chloride), the subject completed the 14-day regimen without signs of hemolysis.^133^ However, smaller daily doses of MB administered to G6PD-deficient African children with acute falciparum malaria resulted in slightly more posttreatment hemolysis than in G6PD-normal children.^134^

MB is registered (as Proveblue^®^, Martindale Pharma, Buckinghamshire, United Kingdom) as a solution for injection, 5 mg/mL for the following indication: treatment of medicinal and chemical products–induced methemoglobinemia. Methylthioninium chloride is indicated in adults and children above the age of 3 months. However, it is currently contraindicated in patients with G6PDd due to the risk of hemolytic anemia.

Early stage development indicates some signs of genotoxicity in cell-level gene mutation assays, but not in in vivo murine studies. MB showed carcinogenic potential in male mice and rats. There are no adequate data on the use of methylthioninium chloride in pregnant women, but studies in animals demonstrate reproductive toxicity. The potential risk for humans is unknown. Preliminary work in huSCID/G6PD mice showed no signs of hemolytic toxicity at up to 50 mg/kg (the human experiment mentioned above was 8.3 mg/kg). This compound is under investigation as an 8-aminoquinoline-induced hemolysis-blocking agent.

### Bulaquine.

Bulaquine (BQ) is a prodrug of PQ and is believed to hydrolyze in the stomach to PQ. Clinical trials of BQ have failed to show that this agents is superior to PQ in terms of safety or anti-relapse efficacy for relapse and this role in clinical practice remains limited.^135^,^136^ One study reported BQ to be better than PQ in G6PD-deficient patients but this only included three subjects, and pharmacokinetic analysis was not available.^137^

### Tinidazole.

Tinidazole is a 5-nitroimidazole used for the treatment of amebiasis and giardiasis. In the *Plasmodium cynomolgi*/macaque relapsing malaria model, tinidazole cured one of six macaques studied with an apparent mild delay to relapse in the other five monkeys. The only study in humans was conducted in healthy G6PD-normal Thai adults with *P. vivax* infection. Subjects were randomized to treatment with either 2 g of oral tinidazole daily for 5 days or standard therapy with PQ (30 mg base per day for 14 days); all patients received CQ (at 25 mg/kg/day for 5 days). Six of the first seven subjects treated with tinidazole relapsed before day 63. The authors of this study concluded that tinidazole was ineffective in preventing relapse of *P. vivax* given at 2 g a day for 5 days concurrently with CQ. The macaque relapsing model appeared to predict correctly the outcome in humans.^138^

### Finding new molecules against relapse.

There is no reliable *P. vivax* liver-stage assay currently available to drive drug discovery; hence, the current strategy to identify novel compounds relies largely on a pragmatic approach and the application of surrogate assays. The current approach consists of screening a very large number of compounds in a high-throughput *P. falciparum* blood-stage assay, and selection of the most likely candidates to be tested in a secondary lower throughput assays such as the *Plasmodium yoelii* liver-stage assay.^139^ While liver schizontocidal activity against a rodent malaria is not directly relevant for relapse, it has been used as a tool to prioritize compounds for screening in any low-throughput in vitro relapse assay. To date all compounds blocking relapse have shown to have causal prophylactic activity. A *P. cynomolgi* liver-stage assay^140^ uses *P. cynomolgi* sporozoite infections of primary rhesus hepatocytes to generate, reproducibly, small parasite forms. The latter have been shown to reactivate in vitro, suggesting that they may be hypnozoites.^141^ This assay represents the focus of current hypnozoiticidal drug discovery efforts. Potential compounds identified by the *P. yoelii* assay are then tested further to find series with a better therapeutic ratio than PQ.

A *P. vivax* liver-stage in vitro assay is under development to replace the current model. The aim is to develop assays with a throughput that is sufficient to screen large libraries directly. The development of this assay as well as other tools to aid in the development of anti-relapse molecule have been reviewed elsewhere (see the article by Olliaro and others).

Analysis of over 5 million compounds from approximately 20 compound collections from the pharmaceutical and biotechnology companies, academic diversity collections, and diversity providers, generated around 25,000 compounds with activity against the blood stage of the parasite IC50s ranging from 1 to 3 μM. The liver-stage activity of a selection of those hits was then assessed using the *P. yoelii* assay.^142^ Some of these chemical entities are now high priority for the Medicines for Malaria Venture and its partners, and are entering lead optimization and clinical development (see the following link for review: http://www.mmv.org/research-development/rd-portfolio).

Work has also been undertaken to evaluate the liver-stage activity of the current portfolio of schizonticidal compounds, with more than 50 anti-infectives currently in use or under development. Several of these antimalarials with schizontocidal activity in a *P. yoelii* liver-stage assay are currently being evaluated in the *P. cynomolgi S* assay for their potential effect on hypnozoites.

Progress in trying to identify potential new candidates as anti-relapse agents has been made; it is however important to note that beyond those mentioned here, there are no new compounds in the global malaria portfolio in preclinical development specifically for their anti-relapse activity. The first generation of compounds that have been specifically optimized based on their potential for anti-relapse activity is expected to enter preclinical development in 2014.

## Conclusions

The treatment of *P. vivax* has changed little over the last 60 years although recent technological advances promise to deliver better, more sensitive and field-adapted diagnostics for parasitemia and G6PDd, and to identify novel antimalarial compounds. *Plasmodium falciparum* is resistant to CQ in most endemic regions. CQR *P. vivax*, although slower to appear than *P. falciparum*, is spreading throughout the endemic world. A unified treatment policy for malaria caused by any species of *Plasmodium* may offer significant individual and public health advantages over the current strategy that relies on CQ plus PQ. ACTs are highly effective against *P. vivax*, and the more slowly slowly-eliminated ACTs suppress the early relapses, but whether this reduces transmission in the long term remains to be proven. The most important potential chemotherapeutic means of interrupting transmission of vivax malaria will be radical cure and thus prevention of all future relapses by using a safe and well-tolerated hypnozoitocidal medication. Primaquine is the only licensed hypnozoitocidal drug, but studies are underway to find more practical ways of deploying it. Renewed efforts are underway to develop alternative treatments for safer and more effective radical cure.

## Key Points

- A unified treatment policy for malaria of any parasitological cause will confer significant individual, public health, and operational benefits in regions co-endemic for *P. falciparum* and *P. vivax*.
- Slowly eliminated ACTs offer posttreatment prophylaxis against the first relapse of tropical strains of *P. vivax*, which in the short term reduces the risk of anemia and the transmissibility of the parasite.
- Ensuring adherence to a complete course of PQ is the greatest challenge in the control of *P. vivax* malaria, and may be achieved by adopting short-course, high-dose regimens, although the safety of these regimens has not been established.
- A robust bedside test for G6PD deficiency is urgently needed to define the toxicity profile of PQ and facilitate the safe deployment of anti-relapse therapy.

## Supplementary Material

Supplemental Annexes.

## Figures and Tables

**Table 1:** Top scoring rapid diagnostic test brands for diagnosis of acute vivax malaria*

Brand	Manufacturer	Product catalog no.
CareStart kits (5)	AccessBio, Inc.	G0111, GO121, GO131, G0161, GO171
SD Bioline kits (4)	Standard Diagnostics, Inc.	05FK60, 05FK66, 05FK80, 05FK100
BIONOTE Malaria P.f. and P.v. Ag Rapid Test Kit	Bionote, Inc.	RG19-12
Humasis Malaria P.f/P.v Antigen Test	Humasis Co. Ltd.	AMFV-7025
NanoSign Malaria PF/Pan Ag 3.0	Bioland Ltd.	RMAP-10

* Parasite detection score > 90%, false positive rate < 10%, error rate < 10% for both *Plasmodium falciparum* and *Plasmodium vivax* at 200 parasites/μL in Round 4 of the World Health Organization evaluation of these devices.^10^

**Table 2:** G6PDd validated quantitative assays and the commercial providers

Kit name	Manufacturer	Venipuncture	Test readout	Chemical basis	Cold storage	Laboratory equipment/skills	Cost/test
G6PD deficiency	Trinity Biotech, Ireland	Yes	Quantitative	Spectrophotometric	Yes	Yes	
G-Six Kinetic	Tulip Group, India	Yes	Quantitative	Spectrophotometric	Yes	Yes	$1.10
R&D G6PD quantitative	N. Dimopoulos S.A., Greece	Optional	Quantitative	Spectrophotometric	Yes	Yes	$0.26–2.50*
R&D G6PD qualitative	N. Dimopoulos S.A., Greece	Optional	Qualitative	Ultra-violet fluorescence	Yes	Yes	$0.18
G6PD-WST	Dojindo, Japan	Optional	Qualitative	Dye reduction	Yes	Yes	$2.00
G-SIX	Tulip Group, India	Yes	Qualitative	Met-Hb reduction	No	Yes	$1.12
G6PD deficiency	Trinity Biotech, Ireland	Yes	Qualitative	Ultra-violet fluorescence	Yes	Yes	
G6PD deficiency	Trinity Biotech, Ireland	Yes	Qualitative	Dye reduction	Yes	Yes	
MBK	Span, India	Yes	Qualitative	Dye reduction	Yes	Yes	
BinaxNOW G6PD	Alere/Inverness Medical, United States	Yes	Qualitative	Dye reduction	No	No	$16.00
CareStart G6PD	AccessBio, United States	No	Qualitative	Dye reduction	No	No	$1.50

* Depends upon numbers of assays run simultaneously, numbers of kits purchased, suppliers of the kits, or use of reagents in lieu of kits.

**Table 3:** Common sources for misdiagnosis of CQR and CQS *Plasmodium vivax*

	Explanation	Recommendation
Incorrect diagnosis of CQS		
Enrollment of patients without clinical disease	Host immunity in asymptomatic patients enrolled from cross-sectional surveys, may facilitate clearance of parasitaemia even following partially effective drug treatment	Restrict efficacy trials to patients presenting with clinical disease
Coadministration of early PQ	Early PQ has schizontocidal activity that can increase parasite clearance and prevent recrudescent infections	Primaquine treatment should be delayed until the end of the follow-up period
Short duration of follow-up	Early evidence of resistance is manifest by late recrudescence	Patients should be followed up for a minimum of 28 days
Incorrect diagnosis of CQR		
Incomplete treatment course	From poor patient adherence	Supervision of drug treatment
Dose of chloroquine administered too low	Prescription of inadequate mg/kg dose	Documentation of exact dose of drug administered
Poor absorption of drug	Either from poor quality drug, reduced gastrointestinal absorption	Measurement of drug blood concentrations on day 7 and the day of parasite recurrence
Poor drug quality	Faulty product	Confirmation of adequate drug levels, pharmacologic evaluation of study drugs, and purchase only from certified, trusted producers
Inadequate sample size	Leading to wide confidence intervals of high failure rates derived from very few cases	Recruitment of an adequate sample (> 70 patients)

CQR = chloroquine resistant; CQS = chloroquine sensitive; PQ = primaquine.
