# Supplementary material for: Diagnosis and Treatment of Plasmodium vivax Malaria
Source: Am J Trop Med Hyg. 2016 Dec 28;95(6 Suppl):35–51. doi: 10.4269/ajtmh.16-0171 (PMC5198890; doi:10.4269/ajtmh.16-0171)
Supplement: Supplementary file 1 [file SD1.pdf]

Supplemental Annex A

Trials of antimalarials against erythrocytic stages of *Plasmodium vivax* infection from 1980 to 2013 (supplemental to Price and others[72])

First author	Year	Title	Journal	Volume	Issue	Page	PubMed ID
Abdallah	2012	Efficacy of artemether-lumefantrine as a treatment of uncomplicated *Plasmodium vivax* malaria in eastern Sudan	*Malaria J*	11	1	404	23217037
Adak	2001	*Plasmodium vivax* polymorphism in a clinical drug trial	*Clin Diagn Laboratory Immunol*	8	5	891–894	11527798
Alcantara	1985	A comparative clinical study of mefloquine and chloroquine in the treatment of vivax malaria	*Southeast Asian J Trop Med Public Health*	16	4	535–539	3915155
Anez	2012	Therapeutic response of *Plasmodium vivax* to chloroquine in Bolivia [in Spanish]	*Biomedica*	32	4	527–535	23715228
Awab	2010	Dihydroartemisinin-piperaquine vs. chloroquine to treat vivax malaria in Afghanistan: an open randomized, non-inferiority, trial	*Malaria J*	9	1	105	20409302
Baird	1995	Treatment of chloroquine-resistant *Plasmodium vivax* with chloroquine and primaquine or halofantrine	*J Infect Dis*	171		1678–1682	7769318
Baird	1997	In vivo resistance to chloroquine by *Plasmodium vivax* and *Plasmodium falciparum* at Nabire, Irian Jaya, Indonesia	*Am J Trop Med Hyg*	56	6	627–631	9230793
Baird	2002	Chloroquine for the treatment of uncomplicated malaria in Guyana	*Ann Trop Med Parasitol*	96	4	339–348	12171615
Baird	1996a	Chloroquine sensitive *Plasmodium falciparum* and *P. vivax* in Central Java, Indonesia	*Trans R Soc Trop Med Hyg*	90		412–413	8882191
Baird	1996b	Survey of resistance to chloroquine by *Plasmodium vivax* in Indonesia	*Trans R Soc Trop Med Hyg*	90		409–411	8882190
Baird	1996c	Survey of resistance to chloroquine of falciparum and vivax malaria in Palawan, The Philippines	*Trans R Soc Trop Med Hyg*	90		413–414	8882192
Barnadas	2008a	*Plasmodium vivax* resistance to chloroquine in Madagascar: clinical efficacy and polymorphisms in *pvmdr1* and *pvcrt-o* genes	*Antimicrob Agents Chemother*	52	12	4233–4240	18809933
Barnadas	2008b	*Plasmodium vivax dhfr* and *dhps* mutations in isolates from Madagascar and therapeutic response to sulphadoxine-pyrimethamine	*Malaria J*	7	35	35	18302746
Castillo	2002	Assessment of therapeutic response of *Plasmodium vivax* and *Plasmodium falciparum* to chloroquine in a Malaria transmission free area in Colombia	*Mem Inst Oswaldo Cruz*	97	4	559–562	12118291
Congpuong	2002	Sensitivity of *Plasmodium vivax* to chloroquine in Sa Kaeo Province, Thailand	*Acta Trop*	83		117–121	12088852
Congpuong	2011	In vivo sensitivity monitoring of chloroquine for the treatment of uncomplicated vivax malaria in four bordered provinces of Thailand during 2009–2010	*J Vector Borne Dis*	48	4	190–196	22297279
Dao	2007	Vivax malaria: preliminary observations following a shorter course of treatment with artesunate plus primaquine	*Trans R Soc Trop Med Hyg*	101		534–539	17368694
Darlow	1982	Sulfadoxine-pyrimethamine for the treatment of acute malaria in children of Papua New Guinea. II. *Plasmodium vivax*	*Am J Trop Med Hyg*	31	1	10–13	7036765
de Santana Filho	2007	Chloroquine-resistant *Plasmodium vivax*, Brazilian Amazon	*Emerg Infect Dis*	13	7	1125–1127	18214203
Dilmec	2010	Monitoring of failure of chloroquine treatment of *Plasmodium vivax* using polymerase chain reaction in Sanliurfa province, Turkey	*Parasitol Res*	106		783–788	20140453

(continued)

SUPPLEMENTAL ANNEX A
Continued

First author	Year	Title	Journal	Volume	Issue	Page	PubMed ID
Dixon	1985	A clinical trial of mefloquine in the treatment of *Plasmodium vivax* malaria.	*Am J Trop Med Hyg*	34	3	435–437	3890575
Dunne	2005	A double blind, randomized study of azithromycin compared with chloroquine for the treatment of *Plasmodium vivax* malaria	*Am J Trop Med Hyg*	73	6	1108–1111	16354821
Ebisawa	1986	A combination of sulfamonomethoxine and pyrimethamine vs. other drugs for the treatment of malaria	*Jpn J Exp Med*	56	5	213–219	3543442
Eibach	2012	Therapeutic efficacy of artemether-lumefantrine for *Plasmodium vivax* infections in a prospective study in Guyana	*Malaria J*	11		e347	23083017
Fryauff	1997	Survey of in vivo sensitivity to chloroquine by *Plasmodium falciparum* and *P. vivax* in Lombok, Indonesia	*Am J Trop Med Hyg*	56	2	241–244	9080887
Fryauff	1999	In vivo responses to antimalarials by *Plasmodium falciparum* and *Plasmodium vivax* from isolated Gag Island off northwest Irian Jaya, Indonesia	*Am J Trop Med Hyg*	60	4	542–546	10348226
Fryauff	2002	The drug sensitivity and transmission dynamics of human malaria on Nias Island, North Sumatra, Indonesia	*Ann Trop Med Parasitol*	96	5	447–462	12194705
Fryauff	1998a	Chloroquine-resistant *Plasmodium vivax* in transmigration settlements of West Kalimantan, Indonesia	*Am J Trop Med Hyg*	4	513–518	9790420	
Fryauff	1998b	Survey of resistance in vivo to chloroquine of *Plasmodium falciparum* and *P. vivax* in North Sulawesi, Indonesia	*Trans R Soc Trop Med Hyg*	92		82–83	9692162
Ganguly	2013	In vivo therapeutic efficacy of chloroquine alone or in combination with primaquine in vivax malaria in Kolkata, West Bengal, India and polymorphism in *pvmdr1* and *pvcrt-0* genes	*Antimicrob Agents Chemother*	57	3	1246–1251	23262997
Genton	2005	Parasitological and clinical efficacy of standard treatment regimens against *Plasmodium falciparum*, *P. vivax* and *P. malariae* in Papua New Guinea	*P N G Med J*	48	3	141–150	17212060
Gogtay	1999	Efficacy of 5- and 14-day primaquine regimen in the prevention of relapses in *Plasmodium vivax* infections	*Ann Trop Med Parasitol*	93		809–812	10715673
Guthmann	2008	*Plasmodium vivax* resistance to chloroquine in Dawei, southern Myanmar	*Trop Med Int Health*	13		91–98	18291007
Hamedi	2002	*Plasmodium vivax* malaria in southeast Iran in 1999–2001: establishing the response to chloroquine in vitro and in vivo	*Southeast Asian J Trop Med Public Health*	33	3	512–517	12693585
Hamedi	2004	Therapeutic efficacy of artesunate in *Plasmodium vivax* malaria in Thailand	*Southeast Asian J Trop Med Public Health*	35	3	570–574	15689068
Hapuarachchi	2004	Chloroquine resistant falciparum malaria among security forces personnel in the Northern Province of Sri Lanka	*Ceylon Med J*	49	2	47–51	15334798
Harinasuta	1985	Trials of mefloquine in vivax and of mefloquine plus 'fansidar' in falciparum malaria	*Lancet*	8434		885–888	2858743
Hasugian	2007	Dihydroartemisinin-piperaquine vs. artesunate-amodiaquine: superior efficacy and posttreatment prophylaxis against multidrug-resistant *Plasmodium falciparum* and *Plasmodium vivax* malaria	*Clin Infect Dis*	44		1067–74	17366451

(continued)

Supplemental Annex A
Continued

First author	Year	Title	Journal	Volume	Issue	Page	PubMed ID
Hasugian	2009	In vivo and in vitro efficacy of amodiaquine monotherapy for treatment of infection by chloroquine-resistant *Plasmodium vivax*	*Antimicrob Agents Chemother*	53	3	1094–1099	19104023
Heidari	2012	In vivo susceptibility of *Plasmodium vivax* to chloroquine in southeastern Iran	*Iran J Parasitol*	7	2	81–84	23109940
Hwang	2013	In vivo efficacy of artemether-lumefantrine and chloroquine against *Plasmodium vivax*: a randomized open label trial in central Ethiopia	*PLoS One*	8	5	e63433	23717423
Jagota	1993	Halofantrine in the treatment of acute malaria: a multi-center study in 268 patients	*Curr Med Res Opin*	13	3	140–144	8222741
Kaneko	1999	Intrinsic efficacy of proguanil against falciparum and vivax malaria independent of the metabolite cycloguanil	*J Infect Dis*	179		974–979	10068594
Karunajeewa	2008	A trial of combination antimalarial therapies in children from Papua New Guinea.	*N Engl J Med*	359	24	2545	19064624
Ketema	2009	Chloroquine-resistant *Plasmodium vivax* malaria in Serbo town, Jimma zone, south-west Ethiopia	*Malaria J*	8		177	19642976
Ketema	2011	Therapeutic efficacy of chloroquine for treatment of *Plasmodium vivax* malaria cases in Halaba district, south Ethiopia	*Parasit Vectors*	4	46	1–7	21453465
Khan	2006	Efficacy and safety of halofantrine in Pakistani children and adults with malaria caused by *P. falciparum* and *P. vivax*	*Southeast Asian J Trop Med Public Health*	37	4	613–618	17121283
Kinzer	2010	Active case detection, treatment of falciparum malaria with combined chloroquine and sulphadoxine/pyrimethamine and vivax malaria with chloroquine and molecular markers of antimalarial resistance in the Republic of Vanuatu	*Malaria J*	9		89	20370920
Kolaczinski	2007	Sulfadoxine-pyrimethamine plus artesunate compared with chloroquine for the treatment of vivax malaria in areas co-endemic for *Plasmodium falciparum* and *P. vivax*: a randomised non-inferiority trial in eastern Afghanistan	*Trans R Soc Trop Med Hyg*	101		1081–1087	17707447
Krudsood	2007	Clinical efficacy of chloroquine vs. artemether-lumefantrine for *Plasmodium vivax* treatment in Thailand	*Korean J Parasitol*	45	2	111–114	17570973
Kurcer	2004	Efficacy of chloroquine in the treatment of *Plasmodium vivax* malaria in Turkey	*Ann Trop Med Parasitol*	98	5	447–451	15257793
Kurcer	2006	The decreasing efficacy of chloroquine in the treatment of *Plasmodium vivax* malaria, in Sanliurfa, south-eastern Turkey	*Ann Trop Med Parasitol*	100	2	109–113	16492358
Lacy	2002	Atovaquone/proguanil therapy for *Plasmodium falciparum* and *Plasmodium vivax* malaria in Indonesians who lack clinical immunity	*Clin Infect Dis*	35		92–95	12384852
Lal	1982	A comparative trial of oral chloroquine and oral co-trimoxazole in vivax malaria in children	*Am J Trop Med Hyg*	31	3	438–440	7044160
Leang	2013	Efficacy of dihydroartemisinin-piperaquine for the treatment of uncomplicated *Plasmodium falciparum* and *Plasmodium vivax* in Cambodia, 2008–2010	*Antimicrob Agents Chemother*	57	9	818–826	23208711

(continued)

Supplemental Annex A
Continued

First author	Year	Title	Journal	Volume	Issue	Page	PubMed ID
Lee	2009	Biological resistance of hydroxychloroquine for *Plasmodium vivax* malaria in the Republic of Korea	*Am J Trop Med Hyg*	81	4	600–604	19815873
Leslie	2007	Sulfadoxine-pyrimethamine, chlorproguanil-dapsone, or chloroquine for the treatment of *Plasmodium vivax* malaria in Afghanistan and Pakistan: a randomized controlled trial	*JAMA*	297	20	2201–2209	17519409
Leslie	2008	A randomized controlled trial of 8 weeks, once weekly primaquine regimen to prevent relapse of *Plasmodium vivax* in Northwest Frontier Province, Pakistan	*PLoS One*	3	8	2861	18682739
Li	1994	Clinical trials of artemisinin and its derivatives in the treatment of malaria in China	*Trans R Soc Trop Med Hyg*	88		5–6	8053027
Lim	1999	Response to chloroquine of *Plasmodium vivax* among South Korean soldiers	*Ann Trop Med Parasitol*	93	6	565–568	10707102
Liu	2013	Artemisinin-naphthoquine combination vs. chloroquine-primaquine to treat vivax malaria: an open-label randomized and non-inferiority trial in Yunnan Province, China	*Malaria J*	12	409	409	24215565
Llanos-Cuentas	2013	Tafenoquine plus chloroquine for the treatment and relapse prevention of *Plasmodium vivax* malaria (DETECTIVE): a multicentre, double-blind, randomised, phase 2b dose-selection study	*Lancet*	383	9922	1049–1058	24360369
Lon	2014	Efficacy of two vs. three-day regimens of dihydroartemisinin-piperaquine for uncomplicated malaria in military personnel in northern Cambodia: an open-label randomized trial	*PLoS One*	9	3	e93138	24667662
Looareesuwan	1996	Clinical studies of atovaquone, alone or in combination with other antimalarial drugs, for treatment of acute uncomplicated malaria in Thailand	*Am J Trop Med Hyg*	54	1	62–66	8651372
Looareesuwan	1999a	Chloroquine sensitivity of *Plasmodium vivax* in Thailand	*Ann Trop Med Parasitol*	93	3	225–230	10562823
Looareesuwan	1999b	Atovaquone and proguanil hydrochloride followed by primaquine for treatment of *Plasmodium vivax* malaria in Thailand	*Trans R Soc Trop Med Hyg*	93		637–640	10717754
Luxemburger	1999	Treatment of vivax malaria on the western border of Thailand	*Trans R Soc Trop Med Hyg*	93		433–438	10674098
Machado	2003	Correlation between *Plasmodium vivax* variants in Belém, Pará State, Brazil and symptoms and clearance of parasitaemia	*Braz J Infect Dis*	7	3	175–177	14499040
Maguire	2002	Chloroquine or sulfadoxine-pyrimethamine for the treatment of uncomplicated, *Plasmodium falciparum* malaria during an epidemic in Central Java, Indonesia	*Ann Trop Med Parasitol*	96	7	655–668	12537627
Maguire	2006	Mefloquine is highly efficacious against chloroquine-resistant *Plasmodium vivax* malaria and *Plasmodium falciparum* malaria in Papua, Indonesia	*Clin Infect Dis*	42	8	1067–1072	16575721
Marfurt	2007	Low efficacy of amodiaquine or chloroquine plus sulfadoxine-pyrimethamine against *Plasmodium falciparum* and *P. vivax* malaria in Papua New Guinea	*Am J Trop Med Hyg*	77	5	947–954	17984359
Marques	2014	*Plasmodium vivax* chloroquine resistance and anemia in the western Brazilian Amazon	*Antimicrob Agents Chemother*	58	1	342–347	24165179
McGready	2002	The effects of quinine and chloroquine antimalarial treatments in the first trimester of pregnancy	*Trans R Soc Trop Med Hyg*	96	2	180–184	12055810

(continued)

Supplemental Annex A
Continued

First author	Year	Title	Journal	Volume	Issue	Page	PubMed ID
Mishra	2012	Monitoring antimalarial drug resistance in India via sentinel sites: outcomes and risk factors for treatment failure, 2009–2010	*Bull World Health Organ*	90	12	895–904	23284195
Mohapatra	2002	Atypical manifestation of *Plasmodium vivax* malaria	*Indian J Malariol*	39	40940	18–25	14686106
Muhamad	2011	Monitoring of clinical efficacy and in vitro sensitivity of *Plasmodium vivax* to chloroquine in area along Thai Myanmar border during 2009–2010	*Malaria J*	10		44	21324161
Murphy	1993	Vivax malaria resistant to treatment and prophylaxis with chloroquine	*Lancet*	341	8837	96–100	8093414
Nandy	2003	Monitoring the chloroquine sensitivity of *Plasmodium vivax* from Calcutta and Orissa, India	*Ann Trop Med Parasitol*	97	3	215–220	12803853
Nateghpour	2007	Evaluation of sensitivity of *Plasmodium vivax* to chloroquine	*Iran J Public Health*	36	3	60–63	Non-PubMed
Nguyen	1993	Treatment of malaria in Vietnam with oral artemisinin	*Am J Trop Med Hyg*	48	3	398–402	8470777
Osorio	2007	Assessment of the efficacy of antimalarial drugs in Tarapaca, in the Colombian Amazon basin [in Spanish]	*Biomedica*	27	1	133–140	17546230
Phan	2002	Artemisinin or chloroquine for blood stage *Plasmodium vivax* malaria in Vietnam	*Trop Med Int Health*	7	10	858–864	12358621
Phyo	2011	Dihydroartemisinin-piperaquine vs. chloroquine in the treatment of *P. viva*x malaria in Thailand: a randomized controlled trial	*Clin Infect Dis*	53		977–984	22002979
Pinto	2003	Assessment of chloroquine single dose treatment of malaria due to *Plasmodium vivax* in Brazilian Amazon	*Rev Inst Med Trop Sao Paulo*	45	6	327–331	14762633
Poravuth	2011	Pyronaradine-artesunate vs. chloroquine in patients with acute *P. vivax* malaria: a randomized double-blind, non-inferiority trial	*PLoS One*	6	1	14501	21267072
Potkar	1995	Resurgence of malaria and drug resistance in *Plasmodium falciparum* and *Plasmodium vivax* species in Bombay	*J Assoc Physicians India*	43	5	336–338	9081964
Pukrittayakamee	2000	Therapeutic responses to different antimalarial drugs in vivax malaria.	*Antimicrob Agents Chemother*	44		1680–1685	10817728
Pukrittayakamee	2001	Therapeutic responses to antibacterial drugs in vivax malaria	*Trans R Soc Trop Med Hyg*	95		524–528	11706666
Pukrittayakamee	2010	A comparison of two short-course primaquine regimens for the treatment and radical cure of *Plasmodium vivax* malaria in Thailand	*Am J Trop Med Hyg*	82	4	542–547	20348496
Pukrittayakamee	1994a	Blood stage antimalarial efficacy of primaquine in *Plasmodium vivax* malaria	*J Infect Dis*	169		932-935	8133114
Pukrittayakamee	1994b	Antimalarial effects of rifampin in *Plasmodium vivax* malaria	*Antimicrob Agents Chemother*	38	3	551–514	8203846
Rajgor	2003	Efficacy of a 14 day primaquine regimen in preventing relapses in patients with *Plasmodium vivax* malaria in Mumbai, India	*Trans R Soc Trop Med Hyg*	97		438–440	15259476
Rao	1993	Efficacy and safety of halofantrine in acute malaria	*J Assoc Physicians India*	41	8	507-508	8294355
Ratcliff	2007a	Therapeutic response of multidrug-resistant *Plasmodium falciparum* and *P. vivax* to chloroquine and sulfadoxine—pyrimethamine in southern Papua, Indonesia	*Trans R Soc Trop Med Hyg*	101		351–359	17028048
Ratcliff	2007b	Two fixed-dose artemisinin combinations for drug-resistant falciparum and vivax malaria in Papua, Indonesia: an open-label randomised comparison	*Lancet*	369		757–765	17336652

(continued)

Supplemental Annex A
Continued

First author	Year	Title	Journal	Volume	Issue	Page	PubMed ID
Rios	2013	Ten years of chloroquine efficacy for uncomplicated *Plasmodium vivax* malaria treatment, Turbo, Antioquia, 2002 and 2011	*Biomedica*	33	3	429–438	24652179
Rowland	1999	Randomized controlled trials of 5- and 14-days primaquine therapy against relapses of vivax malaria in an Afghan refugee settlement in Pakistan	*Trans R Soc Trop Med Hyg*	93	6	641–643	10717755
Ruebush	2003	Chloroquine-resistant *Plasmodium vivax* malaria in Peru	*Am J Trop Med Hyg*	69	5	548–552	14695094
Saravu	2012	*Plasmodium vivax* remains responsive to chloroquine with primaquine treatment regimen: a prospective cohort study from tertiary care teaching hospital in southern India	*Trop Doc*	42	3	163–164	22516030
Shalini	2014	Chloroquine efficacy studies confirm drug susceptibility of *Plasmodium vivax* in Chennai, India	*Malaria J*	13	1	129	24685286
Silachamroon	2003	Clinical trial of oral artesunate with or without high-dose primaquine for the treatment of vivax malaria in Thailand	*Am J Trop Med Hyg*	69	1	14–18	12932090
Singh	2000	Emergence of chloroquine-resistant vivax malaria in south Bihar (India)	*Trans R Soc Trop Med Hyg*	94		327	10975013
Soto	2001	*Plasmodium vivax* clinically resistant to chloroquine in Colombia	*Am J Trop Med Hyg*	65	2	90–93	11508397
Srivastava	2008	Therapeutic responses of *Plasmodium vivax* and *P. falciparum* to chloroquine, in an area of western India where *P. vivax* predominates	*Ann Trop Med Parasitol*	102	66	471–480	18782486
Sumawinata	2003	Very high risk of therapeutic failure with chloroquine for uncomplicated *Plasmodium falciparum* and *P. vivax* malaria in Indonesian Papua	*Am J Trop Med Hyg*	68	4	416–420	12875290
Sutanto	2009	Resistance to chloroquine by *Plasmodium vivax* at Alor in the Lesser Sundas Archipelago in eastern Indonesia	*Am J Trop Med Hyg*	81	2	338–342	19635895
Sutanto	2010	Evaluation of chloroquine therapy for vivax and falciparum malaria in southern Sumatra, western Indonesia	*Malaria J*	9	52		20152016
Tan	1995	Clinical response and susceptibility in vitro of *Plasmodium vivax* to the standard regimen of chloroquine in Thailand	*Trans R Soc Trop Med Hyg*	89		426–429	7570887
Tasanor	2006	Clinical-parasitological response and in-vitro sensitivity of *Plasmodium vivax* to chloroquine and quinine on the western border of Thailand	*Trans R Soc Trop Med Hyg*	100		410–418	16497347
Taylor	2000	Assessing drug sensitivity of *Plasmodium vivax* to halofantrine or choroquine in southern, central Vietnam using an extended 28-day in vivo test and polymerase chain reaction genotyping	*Am J Trop Med Hyg*	62	6	693–697	11304056
Taylor	2001	Chloroquine/doxycycline combination vs. chloroquine alone, and doxycycline alone for the treatment of *Plasmodium falciparum* and *Plasmodium vivax* malaria in northeastern Irian Jaya, Indonesia	*Am J Trop Med Hyg*	64	5	223–228	11463107
Teka	2008	Chloroquine-resistant *Plasmodium vivax* malaria in Debre Zeit, Ethiopia	*Malaria J*	7	220	1–8	18959774
Than	1995	Development of resistance to chloroquine by *Plasmodium vivax* in Myanmar	*Trans R Soc Trop Med Hyg*	89		307–308	7660445
Tjitra	2002	Therapeutic efficacies of artesunate-sulfadoxine-pyrimethamine and chloroquine-sulfadoxine-pyrimethamine in vivax malaria pilot studies: relationship to *Plasmodium vivax dhfr* mutations	*Antimicrob Agents Chemother*	46	12	3947–3953	12435700

(continued)

Supplemental Annex A
Continued

First author	Year	Title	Journal	Volume	Issue	Page	PubMed ID
Tjitra	2012	Efficacy and safety of artemisinin-naphthoquine vs. dihydroartemisinin-piperaquine in adult patients with uncomplicated malaria: a multi-center study in Indonesia	*Malaria J*	1		e153	22554203
Trukillo	2002	Adequate clinical and parasitological *Plasmodium vivax* response to chloroquine in Colombia (Turbo, Antioquia), 2001 [in Spanish]	*Infectio*	6	1	21–26	Non-PubMed
Tulu	1996	Failure of chloroquine treatment of malaria in the highlands of Ethiopia	*Trans R Soc Trop Med Hyg*	90		556–557	8944273
Valecha	2006	Therapeutic efficacy of chloroquine in *Plasmodium vivax* from areas with different epidemiological patterns in India and their *Pvdhfr* gene mutation pattern	*Trans R Soc Trop Med Hyg*	100		831–837	16513151
Valibayov	2003	Clinical efficacy of chloroquine followed by primaquine for *Plasmodium vivax* treatment in Azerbaijan	*Acta Trop*	88		99–102	12943984
Vijaykadga	2004	Assessment of therapeutic efficacy of chloroquine for vivax malaria in Thailand	*Southeast Asian J Trop Med Public Health*	35	3	566–569	15689067
Villalobos	2000	In-vivo sensitivity of *Plasmodium vivax* isolates from Rond nia (western Amazon region, Brazil) to regimens including chloroquine and primaquine	*Ann Trop Med Parasitol*	94	8	749–758	11214093
Walsh	1999	Randomized dose-ranging study of the safety and efficacy of WR 238605 (tafenoquine) in the prevention of relapse of *Plasmodium vivax* malaria in Thailand	*J Infect Dis*	180	4	1282–1287	10479159
Walsh	2004	Randomized trial of 3-dose regimens of tafenoquine (WR238605) vs. low-dose primaquine for preventing *Plasmodium vivax* malaria relapse	*Clin Infect Dis*	39		1095–1103	15486831
Wilairatana	1999	Efficacy of primaquine regimens for primaquine-resistant *Plasmodium vivax* malaria in Thailand	*Am J Trop Med Hyg*	61	6	973–977	10674681
Yadav	2002	Radical curative efficacy of 5 day regimen of primaquine for treatment *Plasmodium vivax* malaria in India	*J Parasitol*	88	5	1042–1044	12435158
Yeramian	2005	Efficacy of DB289 in Thai patients with *Plasmodium vivax* or acute, uncomplicated *Plasmodium falciparum* infections	*J Infect Dis*	192	2	319–322	15962227
Yeshiwondim	2010	Therapeutic efficacy of chloroquine and chloroquine plus primaquine for the treatment of *Plasmodium vivax* in Ethiopia	*Acta Trop*	113		105–113	19835832
Yohannes	2011	Confirmed vivax resistance to chloroquine and effectiveness of artemether-lumefantrine for the treatment of vivax malaria in Ethiopia	*Am J Trop Med Hyg*	84	1	137–140	21212216
Zhu	2013	Blood stage of *Plasmodium vivax* in central China is still susceptible to chloroquine plus primaquine combination therapy	*Am J Trop Med Hyg*	89	1	184–187	23669232

Supplemental Annex B

Trials of different doses of primaquine and control groups from clinical trials conducted from 1953 to 2011 (supplemental to John and others[104])

First author	Year of publication	Country	Partner drug	Duration of treatment (days)	Total primaquine dose (mg/kg)	Primaquine supervision	Duration of follow-up (days)	Sample size	Recurrence rate (%) of *Plasmodium vivax* at the end of the study
Very low-dose primaquine (≤ 2.5 mg/kg total dose)									
Baird	1995	Indonesia	Chloroquine	3	2.50	Not stated	28	19	15.8
Dixon	1985	Thailand	Chloroquine	5	1.25	Not stated	28	40	0.0
Krudsood	2008	Thailand	Artesunate	5	2.50	Not stated	28	60	15.0
Di Lorenzo	1953	United States, Korea	Chloroquine	7	1.75	Not stated	90	31	3.0
Villalobos-Salcedo	2000	Brazil	Chloroquine	5	2.50	All doses	90	30	20.0
Alving	1960	United States, Chesson	Chloroquine	28	2.00	Not stated	120	41	90.0
Carmona-Fonseca	2009	Columbia	Chloroquine	3	1.75	All doses	120	26	50.0
Carmona-Fonseca	2009	Columbia	Chloroquine	3	2.50	All doses	120	26	57.7
Abdon	2001	Brazil	Chloroquine	5	2.50	All doses	180	40	20.0
Alvarez	2006	Columbia	Chloroquine	7	1.75	All doses	180	62	41.9
Alvarez	2006	Columbia	Chloroquine	3	0.75	All doses	180	65	49.2
Bergonzoli	2000	Nicaragua, Costa Rica	Chloroquine	5	2.00	All doses	180	30	0.0
Bergonzoli	2000	Nicaragua, Costa Rica	Chloroquine	1	0.75	All doses	180	25	4.0
Da Silva	2003	Brazil	Chloroquine	5	2.50	Not stated	180	26	15.4
Da Silva	2003	Brazil	Artesunate (100 mg)	5	2.50	Not stated	180	25	16.0
Da Silva	2003	Brazil	Artesunate (200 mg)	5	2.50	Not stated	180	20	25.0
Da Silva	2003	Brazil	Artesunate (150 mg)	5	2.50	Not stated	180	20	25.0
Fernandopulle	2003	Sri Lanka	Not stated	5	1.25	All doses	180	6	83.3
Gogtay	1998	India	Chloroquine	5	1.25	All doses	180	100	15.0
Gogtay	1999	India	Chloroquine	5	1.25	All doses	180	62	25.8
Saint-Yves	1977	PNG	Chloroquine	1	0.75	All doses	180	9	67.0
Contacos	1973	United States, West Pakistan	Chloroquine	5	1.25	Not stated	200	5	100.0
Singh	1990	India	Chloroquine	5	1.25	Not stated	240	995	13.2
Cedillos	1978	El Salvador	Amodiaquine	5	1.25	Part supervised	270	90	21.1
Cedillos	1978	El Salvador	Amodiaquine	1	0.75	Part supervised	270	67	23.9
Mendoza	1963	Mexico	Chloroquine	5	1.25	All doses	270	389	20.6
Rowland	1999	Pakistan	Chloroquine	5	1.25	All doses	300	250	51.2
Cooper	1953	United States, Chesson	Quinine	14	2.33	Not stated	350	34	65.0
Cooper	1953	United States, Chesson	Chloroquine	7	2.33	Not stated	350	10	80.0
Thaeler	1953	Nicaragua	Chloroquine	14	2.33	Not stated	360	121	0.0
Adak	2001	India	Chloroquine	5	1.25	All doses	365	220	26.8
Appavoo	1984	India	Chloroquine	3	1.25	Not stated	365	425	3.8
Prasad	1991	India	Chloroquine	5	1.25	All doses	365	883	2.0
Roy	1977	India	Chloroquine	5	1.25	Not stated	365	6,393	1.3
Roy	1979	India	Chloroquine	5	1.25	All doses	365	1,389	0.7
Sharma	1973	India	Chloroquine	5	1.25	All doses	365	140	9.3
Yadav	2002	India	Chloroquine	5	1.25	Not stated	365	759	6.5
Sinha	1989	India	Chloroquine	5	1.25	All doses	395	725	6.9
Basavaraj	1960	India	Chloroquine	5	1.25	Not stated	480	563	6.0
Dua	2001	India	Chloroquine	5	1.25	Not stated	540	5,541	9.2
Srivastava	1996	India	Chloroquine	5	1.25	Not stated	540	173	5.8
Prasad	1991	India	Chloroquine	5	1.25	All doses	720	1,439	4.0
Prasad	1991	India	Chloroquine	5	1.25	All doses	1,080	2,484	5.7
Prasad	1991	India	Chloroquine	5	1.25	All doses	1,440	8,914	23.2
Low-dose primaquine (> 2.5 to < 5.0 mg/kg total dose)									
Looareesuwan	1999a	Thailand	Chloroquine	14	3.5	All doses	28	441	0.5
Singh	2000	India	Chloroquine	14	3.5	Part supervised	28	75	16.0
Buchachart	2001	Thailand	Chloroquine	14	3.5	Not stated	28	364	0.0

(continued)

Supplemental Annex B

Continued

First author	Year of publication	Country	Partner drug	Duration of treatment (days)	Total primaquine dose (mg/kg)	Primaquine supervision	Duration of follow-up (days)	Sample size	Recurrence rate (%) of *Plasmodium vivax* at the end of the study
Congpuong	2002	Thailand	Chloroquine	14	3.5	Not stated	28	26	0.0
Hamedi	2002	Iran	Chloroquine	14	3.5	All doses	28	39	0.0
Valibayov	2003	Azerbaijan	Chloroquine	14	3.5	Not stated	28	143	0.0
Hamedi	2004	Thailand	Artesunate	14	3.5	All doses	28	42	4.8
Yeramian	2005	Thailand	DB289	14	3.5	Not stated	28	9	11.1
Krudsood	2006	Thailand	Chloroquine	7	3.5	All doses	28	68	1.5
Maguire	2006	Indonesia	Mefloquine	14	3.5	All doses	28	310	1.3
Maguire	2006	Indonesia	Chloroquine	14	3.5	All doses	28	249	21.2
Tasanor	2006	Thailand	Chloroquine	14	3.5	All doses	28	24	0.0
Tasanor	2006	Thailand	Quinine	14	3.5	All doses	28	23	0.0
Krudsood	2007	Thailand	Chloroquine	14	3.5	Not stated	28	42	0.0
Krudsood	2007	Thailand	Artemether–lumefantrine	14	3.5	Not stated	28	38	2.6
Krudsood	2008	Thailand	Artesunate	9	4.5	Not stated	28	56	4.0
Krudsood	2008	Thailand	Artesunate	7	3.5	Not stated	28	57	11.0
Lee	2009	Republic of Korea	Chloroquine	14	3.5	Part supervised	28	108	0.0
Pukrittayakamee	2010	Thailand	None	7	3.5	Not stated	28	31	30.0
Pukrittayakamee	1994b	Thailand	Chloroquine	14	3.5	Not stated	28	25	0.0
Pukrittayakamee	1994b	Thailand	None	14	3.5	Not stated	28	30	10.0
Pukrittayakamee	2000	Thailand	Chloroquine	14	3.5	Not stated	28	22	0.0
Pukrittayakamee	2000	Thailand	None	14	3.5	Not stated	28	26	11.5
Pukrittayakamee	1994a	Thailand	Chloroquine	14	3.5	Not stated	30	20	0.0
Pukrittayakamee	1994a	Thailand	Rifampicin	14	3.5	Not stated	30	20	5.0
Machado	2003	Brazil	Chloroquine	14	3.5	Not stated	30	30	0.0
Muhamed	2011	Thailand	Chloroquine	14	3.5	Part supervised	42	130	0.0
Luxemburger	1999	Thailand	Chloroquine	14	3.5	All doses	63	43	7.0
Villalobos-Salcedo	2000	Brazil	Chloroquine	14	3.5	All doses	90	31	6.5
Takeuchi	2010	Thailand	Chloroquine	14	3.5	All doses	90	90	3.3
Takeuchi	2010	Thailand	Chloroquine	14	3.5	Unsupervised	90	97	12.4
Maneeboonyang	2011	Thailand	Chloroquine	14	3.5	All doses	90	43	0.0
Maneeboonyang	2011	Thailand	Chloroquine	14	3.5	Unsupervised	90	33	15.1
Tan-ariya	1995	Thailand	Chloroquine	14	3.5	Part supervised	100	50	12.0
Alving	1953	United States, Korea	Chloroquine	14	3.5	Not stated	120	348	0.0
Alving	1960	United States, Chesson	Chloroquine	14	3.5	Not stated	120	60	27.0
Alving	1960	United States, Chesson	Chloroquine	28	4.0	Not stated	120	20	30.0
Alving	1960	United States, Chesson	Chloroquine	28	3.0	Not stated	120	15	40.0
Alving	1960	United States, Chesson	Chloroquine	56	4.0	Not stated	120	61	55.7
Carmona-Fonseca	2009	Columbia	Chloroquine	14	3.5	All doses	120	66	15.2
Carmona-Fonseca	2009	Columbia	Chloroquine	3	3.5	All doses	120	63	58.7
Yeshiwondim	2010	Ethiopia	Chloroquine	14	3.5	All doses	157	132	3.0
Walsh	2004	Thailand	Chloroquine	14	3.5	All doses	168	12	25.0
Coatney	1953	United States, Korea	None	14	3.5	Not stated	180	294	0.0
Martelo	1969	United States, Vietnam	Chloroquine	14	3.5	Not stated	180	21	14.3
Fisher	1970	United States, Vietnam	Chloroquine	14	3.5	All doses	180	133	7.5
Gogtay	1999	India	Chloroquine	14	3.5	All doses	180	63	0.0
Bergonzoli	2000	Nicaragua, Costa Rica	Chloroquine	14	3.5	All doses	180	26	0.0
Bergonzoli	2000	Nicaragua, Costa Rica	Chloroquine	9	2.8	All doses	180	28	3.6
Abdon	2001	Brazil	Chloroquine	7	3.5	All doses	180	39	0.0
Abdon	2001	Brazil	Chloroquine	14	3.5	All doses	180	40	5.0

(continued)

Supplemental Annex B

Continued

First author	Year of publication	Country	Partner drug	Duration of treatment (days)	Total primaquine dose (mg/kg)	Primaquine supervision	Duration of follow-up (days)	Sample size	Recurrence rate (%) of *Plasmodium vivax* at the end of the study
Duarte	2001	Brazil	Chloroquine	14	3.5	Part supervised	180	50	14.0
Da Silva	2003	Brazil	Artesunate (100 mg)	7	3.5	Not stated	180	22	0.0
Da Silva	2003	Brazil	Chloroquine	7	3.5	Not stated	180	25	4.0
Da Silva	2003	Brazil	Artesunate (200 mg)	7	3.5	Not stated	180	20	5.0
Da Silva	2003	Brazil	Artesunate (150 mg)	7	3.5	Not stated	180	24	20.8
Rajgor	2003	India	Chloroquine	14	3.5	All doses	180	103	5.8
Alvarez	2006	Columbia	Chloroquine	15	3.5	All doses	180	64	18.8
Mendoza	1963	Mexico	Chloroquine	14	3.5	All doses	270	363	10.2
Miller	1974	United States. Chesson	Chloroquine	14	3.5	Not stated	270	57	3.5
Li	1999	China	Chloroquine	8	3.0	All doses	270	35	22.9
Leslie	2004	Pakistan	Chloroquine	14	3.5	All doses	270	210	19.0
Leslie	2004	Pakistan	Chloroquine	14	3.5	All doses	270	173	19.7
Smoak	1997	United States, Somalia	Chloroquine/quinine	14	3.5	Part supervised	300	32	25.0
Smoak	1997	United States, Somalia	Chloroquine/quinine	14	3.5	Part supervised	300	28	64.2
Rowland	1999	Pakistan	Chloroquine	14	3.5	All doses	300	100	32.0
Orjuela-Sanchez	2009	Brazil	Chloroquine	7	3.5	Part supervised	336	87	71.5
Cooper	1953	United States, Chesson	Quinine	14	4.7	Not stated	350	34	15.0
Cooper	1953	United States, Chesson	Chloroquine	7	3.5	Not stated	350	10	90.0
Orjuela-Sanchez	2009	Brazil	Chloroquine	7	3.5	Part supervised	355	77	36.6
Thaeler	1953	Nicaragua	Chloroquine	14	3.5	Not stated	360	151	0.0
Thaeler	1953	Nicaragua	Chloroquine	14	4.7	Not stated	360	49	0.0
Alving	1955	United States, Chesson	Quinine	14	3.5	Not stated	365	19	5.3
Alving	1955	United States, Chesson	Chloroquine	14	3.5	Not stated	365	19	26.3
Alving	1955	United States, Chesson	None	14	3.5	Not stated	365	19	78.9
Saint-Yves	1977	PNG	Chloroquine	14	3.50	All doses	365	10	0.0
Schwartz	2000	Israel	Chloroquine	14	3.5	Not stated	365	15	33.3
Bunnag	1994	Thailand	Chloroquine	14	3.5	Not stated	540	81	8.6
Jelinek	1995	Germany	Chloroquine	14	3.5	Not stated	540	56	12.5
Fang	1999	Taiwan	Chloroquine	14	3.5	Not stated	540	12	8.3
Moon	2009	Republic of Korea	Chloroquine	14	3.5	Not stated	660	3,881	1.6
Haghdoost	2006	Iran	Chloroquine	14	3.5	Not stated	2,555	12,337	7.4
High-dose primaquine (≥ 5.0 mg/kg total dose)									
Baird	1995	Indonesia	Chloroquine	27	10.0	Not stated	28	26	15.4
Baird	1995	Indonesia	Chloroquine (10 mg/kg)	27	10.0	Not stated	28	23	13.0
Fryauff	1997	Indonesia	Halofantrine	28	10.0	All doses	28	26	0.0
Fryauff	1997	Indonesia	Chloroquine	28	10.0	All doses	28	27	11.1
Wilairatana	1999	Thailand	Artesunate	14	7.0	Not stated	28	15	0.0
Wilairatana	1999	Thailand	Sulfadoxine–pyrimethamine	14	7.0	Not stated	28	19	0.0
Wilairatana	1999	Thailand	None	14	7.0	Not stated	28	17	0.0
Lacy	2002	Indonesia	Atovaquone/proguanil	14	7.0	Not stated	28	16	0.0
Silachamroon	2003	Thailand	Artesunate (5 days)	14	7.0	Not stated	28	142	0.0
Silachamroon	2003	Thailand	Artesunate (7 days)	14	7.0	Not stated	28	157	0.0
Dao	2007	Vietnam	Artesunate	7	5.3	All doses	28	28	3.6
Krudsood	2008	Thailand	Artesunate	11	5.5	Not stated	28	48	0.0
Krudsood	2008	Thailand	Artesunate	14	7.0	Not stated	28	52	0.0
Krudsood	2008	Thailand	Artesunate	7	7.0	Not stated	28	49	4.0
Pukrittayakamee	2010	Thailand	None	7	7.0	Not stated	28	33	6.0
Looareesuwan	1999b	Thailand	Atovaquone/proguanil	14	7.0	Not stated	84	35	5.7

(continued)

Supplemental Annex B

Continued

First author	Year of publication	Country	Partner drug	Duration of treatment (days)	Total primaquine dose (mg/kg)	Primaquine supervision	Duration of follow-up (days)	Sample size	Recurrence rate (%) of *Plasmodium vivax* at the end of the study
Alving	1960	United States, Chesson	Chloroquine	56	8.0	Not stated	120	51	5.9
Alving	1960	United States, Chesson	Chloroquine	56	6.0	Not stated	120	40	10.0
Alving	1960	United States, Chesson	Chloroquine	98	7.0	Not stated	120	30	20.0
Contacos	1974	United States, Chesson	Chloroquine	56	6.0	Not stated	133	10	0.0
Martelo	1969	United States, Vietnam	Chloroquine	56	6.0	Not stated	180	21	28.6
Fisher	1970	United States, Vietnam	Chloroquine	56	6.0	Unsupervised	180	94	22.3
Clyde	1977	United States, Chesson	Chloroquine	7	7.0	All doses	293	11	0.0
Leslie	2008	Pakistan	Chloroquine	14	7.0	All doses	330	54	1.9
Leslie	2008	Pakistan	Chloroquine	56	6.0	All doses	330	68	5.9
Saint-Yves	1977	PNG	Chloroquine	14	5.3	All doses	365	10	0.0
Bunnag	1994	Thailand	Chloroquine	14	5.3	Not stated	540	86	1.2
Kaplan	1974	United States, Somalia	Chloroquine	21	5.3	All doses	895	207	4.3
Primaquine dose not stated									
Dunne	2005	India	Azithromycin	14	Not stated	Not stated	28	100	0.0
Dunne	2005	India	Chloroquine	14	Not stated	Not stated	28	97	0.9
Control arms (no primaquine)									
Dixon	1985	Thailand	Chloroquine	No primaquine			28	11	0.0
Pukrittayakamee	1994b	Thailand	Chloroquine	No primaquine			28	30	0.0
Baird	1995	Indonesia	Chloroquine	No primaquine			28	23	87.0
Baird	1995	Indonesia	Chloroquine	No primaquine			28	22	68.2
Looareesuwan	1999a	Thailand	Chloroquine	No primaquine			28	445	0.5
Pukrittayakamee	2000	Thailand	Chloroquine	No primaquine			28	21	0.0
Walsh	2004	Thailand	Chloroquine	No primaquine			56	10	80.0
Luxemburger	1999	Thailand	Chloroquine	No primaquine			63	69	55.1
Di Lorenzo	1953	United States, Korea	Chloroquine	No primaquine			90	46	50.0
Alving	1953	United States, Korea	Chloroquine	No primaquine			120	355	38.6
Alving	1960	United States, Chesson	Chloroquine	No primaquine			120	74	95.9
Yeshiwondim	2010	Ethiopia	Chloroquine	No primaquine			157	108	8.3
Gogtay	1999	India	Chloroquine	No primaquine			180	60	11.7
Rajgor	2003	India	Chloroquine	No primaquine			180	101	12.9
Leslie	2004	Pakistan	Chloroquine	No primaquine			270	212	40.6
Rowland	1999	Pakistan	Chloroquine	No primaquine			300	250	51.6
Rowland	1999	Pakistan	Chloroquine	No primaquine			300	100	49.0
Leslie	2008	Pakistan	Chloroquine	No primaquine			330	68	32.4
Adak	2001	India	Chloroquine	No primaquine			365	224	40.2
Yadav	2002	India	Chloroquine	No primaquine			365	723	8.6
